# Rationalized Volcano Plot in Heterogeneous Electrochemiluminescence

**DOI:** 10.1021/acselectrochem.6c00072

**Published:** 2026-04-06

**Authors:** Alessandro Fracassa, Michele Orza, Chiara Mariani, Claudio Ignazio Santo, Claudia Martinez Asenjo, Elisa D’Arrigo, Neso Sojic, Francesco Paolucci, Fabrizia Negri, Giovanni Valenti

**Affiliations:** † Department of Chemistry “Giacomo Ciamician”, Alma Mater Studiorum − University of Bologna, Bologna 40129, Italy; ‡ Center for Chemical Catalysis − C3, Alma Mater Studiorum − University of Bologna, Bologna 40129, Italy; § CNRS UMR 5255, Bordeaux INP, ENSMAC, University of Bordeaux, Pessac 33607, France; ∥ CNR-ICMATE, Corso Stati Uniti 4, Padova 35127, Italy

**Keywords:** DFT, radical deprotonation, kinetics, bead-based assay, ECL emitting layer

## Abstract

The performance of electrochemically induced chemiluminescence (ECL) assays strongly depends on the stability of electrogenerated coreactant radicals, which governs the spatial extension of the ECL emitting layer. This dependence is even more pronounced for heterogeneous bead-based ECL, where the emission efficiency is widely assumed to scale proportionally with the thickness of the ECL emitting layer. To date, however, establishing a consistent relationship between ECL intensity and radical stability has remained elusive: apparent rate constants fail to isolate intrinsic deprotonation kinetics, and the rational design of amines with long-lived radical cations remains experimentally challenging. In this work, using ECL microscopy, we address both issues by correlating the ECL intensity from individual [Ru­(bpy)_3_]^2+^-labeled beads with the intrinsic deprotonation kinetics of model electrogenerated radical cations, including a novel long-lived intermediate arising from an aniline derivative. Kinetic constants derived from density functional theory (DFT) calculations, in the framework of a hybrid cluster-continuum approach, disentangled intrinsic radical decomposition rates from experimental artifacts such as concentration, pH, and buffer effects. This combined electrochemical imaging and computational approach reveals a non-monotonic, volcano-type dependence between the deprotonation rate of radical cations and ECL intensity, defining an optimal stability window for efficient ECL emission.

## Graphical abstract

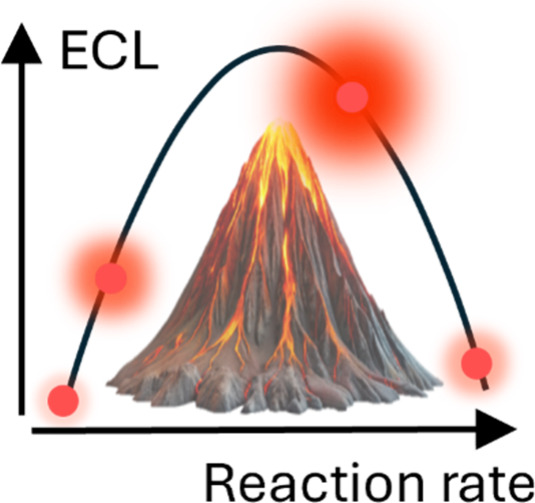

## 1 Introduction

Electrochemical imaging is a spatially resolved approach to precisely map redox reactivity.^1^ The luminescence arising from cascade reactions among electrogenerated radicals, known as electrochemically induced chemiluminescence or electrochemiluminescence (ECL), constitutes an optical means to transduce local redox reactivity into a two-dimensional image via microscopy.^2–6^ Because it resolves emission with sub-micrometric resolution, ECL microscopy (ECLM) overcomes the limitations of ensemble-averaged measurements. Hence, ECLM is currently one of the most powerful tools to access heterogeneous kinetics, facilitating mechanistic understanding of ECL processes in cellular environments or on microbeads surface.^7–9^ Regardless of its ability to spatially resolve ECL processes, ECLM-based kinetic analysis rely on apparent rate constants that alter intrinsic radical reactivity with environmental effects, whereas computational approaches provide access to condition-free kinetics. Bead-based ECL immunoassays represent a leading platform for ultrasensitive clinical diagnostics, counting more than 47,000 Elecsys® instruments installed worldwide.^10–13^ Despite extensive research aimed at understanding the mechanisms governing bead-based ECL generation,^14–22^ the relationship between the stability of electrogenerated radicals and the resulting ECL efficiency remains insufficiently defined. In particular, the kinetics of radical decay, and its impact on light output, requires deeper investigation.

In heterogeneous bead-based systems, [Ru­(bpy)_3_]^2+^ labels are typically immobilized all over the surface of magnetic microbeads (o̷ = 2.8 μm), contrarily to homogeneous ECL where both [Ru­(bpy)_3_]^2+^ and coreactant are free in solution. Such beads have been proven to be an accurate model for that of a heterogeneous bead-based ECL sandwich immunoassay.^15,23,24^ Because of the three-dimensional distribution of the labels on non-conductive microbeads, signal generation in the heterogeneous format relies on the oxidation of sacrificial tertiary amine coreactants, most notably tri-*n*-propylamine (TPrA), which simultaneously yields oxidizing and reducing radicals sufficiently long-lived to excite luminophores beyond the immediate vicinity of the electrode surface.^25^ The diffusion length of the electrogenerated radicals, in particular the radical cation, defines the thickness of the reactive layer along the normal axis to the electrode where excitation of the luminophores occurs by reacting with TPrA radicals (Figure 1). This region, also known as the ECL emission layer thickness (TEL), serves as a descriptor of radical decay kinetics, commonly expressed by a pseudo first-order rate constant.

**Figure 1:**
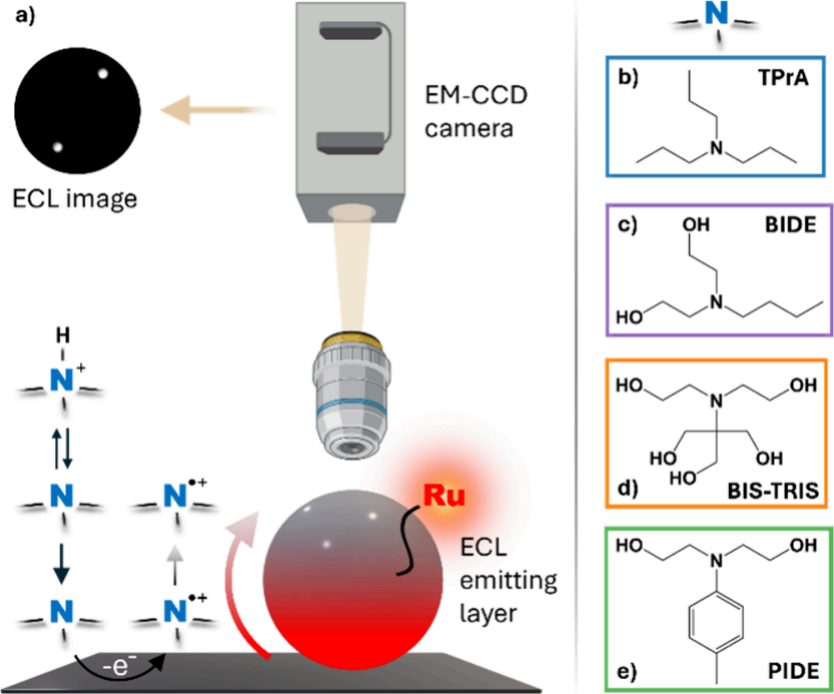
(a) Schematic representation of single-bead ECLM. The thickness of the ECL emitting layer (red gradient to represent [Ru­(bpy)_3_]^2+^ emission) scales with the diffusion layer of the tertiary amine electrogenerated radical cation. Molecular structures labeled N^+^—H, N, and N^•+^ denote the protonated tertiary amine, the deprotonated parent coreactant and its N-centered radical cation, respectively. Instead, Ru represents [Ru­(bpy)_3_]^2+^ covalently attached on the bead surface. Additional mechanistic details are provided in eqs 1–5. The arrows between identical species represent mass transport rather than chemical transformation. The molecular structures reported in the right column represent the coreactants employed throughout this work, namely (b) TPrA, (c) BIDE, (d) BIS-TRIS, and (e) PIDE.

The half-life time of the cation TPrA^•+^ radical in aqueous media was first estimated by Miao et al. by generating ECL at controlled distance with scanning electrochemical microscopy (SECM),^26^ who reported the benchmark pseudo first-order rate constant of 3500 s^–1^. Although the physical hindrance of microbeads rules out the use of SECM in bead-based systems, lateral ECLM represents a powerful alternative to infer the stability of transient species. Once a snapshot of the ECL emitting layer is captured (Figure 1), iterative parametrization of finite element simulations aimed at reproducing experimental ECL profiles enables estimation of radicals stability.^27^ Sentic et al. revealed that the emitting layer in bead-based systems extends as far as 3 μm.^14^ This result is best reproduced with a rate constant of 2920 s^–1^ for TPrA^•+^ deprotonation, in close agreement with the earlier estimate and further supported by recent imaging-based studies across multiple platforms (i.e., microbeads, microelectrodes),^16,18,21,28–30^ as well as by independent interferometric measurements.^22,31,32^

Current understanding assumes that ECL signal intensity from labeled microbeads scales proportionally with the radical cation stability, as longer-lived intermediates can diffuse further and excite more distant luminophores.^32,33^ Recently, this hypothesis was explored in covalent organic framework (COF) nanoemitters, where varying the electron affinity of acrylonitrile linkers produced a volcano-type relationship between ECL intensity and electron affinity.^34^ This comparative investigation suggests that, at least for COF nanoemitters, balanced radical stability is crucial for maximizing ECL efficiency—raising the question of whether a similar principle holds for bead-based ECL systems. This question, however, has never been rigorously addressed, due to both theoretical and experimental challenges. Existing imaging approaches for estimating radical decomposition rates provide only apparent constants that, although consistent with experimental observations, fail to capture the intrinsic reactivity of radical cations. Meanwhile, despite numerous coreactants producing short-lived, highly reactive radicals have been characterized, the rational design of tertiary amines with the opposite behavior has been hindered by the absence of clear molecular guidelines.

Here, the decomposition of a series of tertiary amine radical cations (Figure 1a–d)—TPrA, 2,2′-(butylimino)­diethanol (BIDE), 2,2-bis­(hydroxymethyl)-2,2′,2″-nitrilotriethanol (BIS-TRIS) and 2,2′-(4-methylphenylimino)­diethanol (PIDE), a newly proposed aniline derivative forming an unusually long-lived radical cation—are investigated. These coreactants were selected to span a broad kinetic spectrum, from fast (i.e., BIDE) to slow deprotonation regimes (i.e., BIS-TRIS, PIDE). Their kinetics are disentangled from external contributions by simulating free-energy profiles along deprotonation coordinates via density functional theory (DFT).^35^ The activation barriers are extracted from the computed transition states to derive intrinsic first-order rate constants *k*_H_ (see the Supporting Information), which represent the true kinetics of the radical cation decay, imposed by molecular behavior alone. As such, semi-empirical deprotonation rates establish a physically meaningful framework, independent of experimental artifacts, for correlating ECL emission intensity from single beads with radical cations stability across different coreactants. In this context, spatially resolved ECLM provides direct visualization of individual emission profiles, such that the measured intensity directly reflects the efficiency of the underlying ECL mechanism at the single-bead level. This combined imaging-computational framework reveals, for the first time, a non-monotonic, volcano-type dependence between the deprotonation rate of coreactant radicals and ECL intensity, defining an optimal lifetime window for efficient light generation.

## 2 Experimental Methods

### 2.1 Covalent Functionalization of Amine-Coated Beads

5 μL of beads stock suspension (30 mg·mL^–1^) were washed three times with 200 μL of 0.3 M phosphate buffer (PB, pH 6.8) and <0.1% Tween20 using magnetic support. Subsequently, 150 μL of a 1 mM solution of Ru­(II) complex (5 Ru­(II) complex equivalents per NH_2_) in a 80:20 mixture of 0.3 M PB (pH 6.8) with <0.1% Tween20 and DMSO were added to the beads suspension. The mixture was incubated overnight at room temperature under physical mixing at 1100 rpm by ZX3 Advanced Vortex Mixer (VELP Scientifica Srl, Italy). After incubation, the beads were washed with 200 μL of 0.3 M PB (pH 6.8) with <0.1% Tween20 for 5 times using magnetic support. Finally, Ru­(II)-labeled beads were suspended in 208 μL of 0.3 M PB (pH 6.8) with <0.1% Tween20 to a final concentration of 0.72 mg·mL^–1^ and stored at 4°C.

### 2.2 Computational Methodology

A detailed description of the computational protocol is provided in the Supporting Information. Briefly, quantum chemical calculations were performed using Gaussian 16 and ORCA 6.0.1. An initial conformational search was carried out with the GOAT algorithm at the GFN2-xTB level including implicit solvation, and the lowest-energy conformers were subsequently reoptimized using DFT (M06-2X-D3/def2-SVP, PCM). A hybrid cluster–continuum approach was employed to describe the proton-transfer mechanism, including up to five explicit water molecules to account for specific solute–solvent interactions. Transition states were located and verified through frequency and IRC analyses, and Gibbs free energy barriers (Δ*G* ^‡^) were obtained from the corresponding free energy profiles. Final energies were refined at the M06-2X-D3/def2-TZVP level of theory.

### 2.3 ECL Microscopy

A detailed description of the ECL imaging setup is provided in the Supporting Information. Briefly, measurements were performed in a three-electrode electrochemical cell using GC or Pt working electrodes (*A* = 0.071 cm^2^) onto which Ru­(II)-functionalized microbeads were drop-cast, with a Pt counter electrode and an Ag/AgCl reference electrode. Experiments were carried out in 0.3 M phosphate buffer containing 0.1 M coreactant (TPrA, BIDE, or BIS-TRIS) at pH 6.8, while PIDE was investigated in 0.3 M acetate buffer at pH 5. ECL imaging was performed using an inverted optical microscope equipped with an EMCCD camera under dark conditions. Images were acquired at 200 ms intervals during a double chronoamperometric pulse consisting of 2 s at open circuit potential followed by 1.7 V (vs Ag/AgCl) for 13 s.

### 2.4 Finite Element Simulations

Time-dependent simulations were performed using a 2D axisymmetric model to describe coupled mass transport and reaction processes around a single Ru­(II)-functionalized microbead. The model accounts for diffusion of the coreactant and its reactive intermediates in solution, as well as surface-confined reactions involving [Ru­(bpy)_3_]^2+^ and its redox states. Bulk coreactant concentration was set to 0.1 M, and the reaction scheme includes all key species involved in ECL generation. The full set of electrochemical and chemical reactions, along with the corresponding parameters and rate constants, are provided in the Supporting Information alongside a detailed description.

## 3 Results and Discussion

In this study, 2.8 μm magnetic microbeads were used as scaffolds for the covalent immobilization of [Ru­(bpy)_3_]^2+^, providing a representative model for bead-based ECL immunoassays. Single-bead ECL measurements were carried out at a fixed coreactant concentration of 0.1 M to ensure consistent comparison across different systems. All measurements were performed in 0.3 M phosphate buffer (pH 6.8), except for PIDE, which was dissolved in 0.3 M acetate buffer (pH 5) due to solubility constraints (see Supporting Information for further details). ECL generation in bead-based systems follows the so-called “remote” mechanism,^26^ according to the well-established kinetic model described in eqs 1–5.

$$\mathrm{TPrA}+H_{2}{{\mathrm{PO}}_{4}}^{-}⇌{\mathrm{TPrAH}}^{+}+{{\mathrm{HPO}}_{4}}^{2-}$$ 1

$$\mathrm{TPrA}⇌{\mathrm{TPrA}}^{•+}+e^{-}$$ 2

$${\mathrm{TPrA}}^{•+}⇌{\mathrm{TPrA}}^{•}+H^{+}$$ 3

$${\left[\mathrm{Ru}{\left(\mathrm{bpy}\right)}_{3}\right]}^{2+}+{\mathrm{TPrA}}^{•}\to {\left[\mathrm{Ru}{\left(\mathrm{bpy}\right)}_{3}\right]}^{+}+P$$ 4

$${\left[\mathrm{Ru}{\left(\mathrm{bpy}\right)}_{3}\right]}^{+}+{\mathrm{TPrA}}^{•+}\to {\left[\mathrm{Ru}{\left(\mathrm{bpy}\right)}_{3}\right]}^{2+*}+\mathrm{TPrA}$$ 5

Due to its basic character (p*K*_a_ = 10.4),^36^ TPrA is subjected to a preceding acid–base equilibrium in aqueous solutions (eq 1). In buffered neutral environment it exists predominantly as the protonated ammonium species, TPrAH^+^, leaving only a fraction of the initial concentration to participate in the oxidation process.^37,38^ Upon direct oxidation at the electrode, TPrA evolves into TPrA^•+^ radical cation (eq 2), which subsequently undergoes deprotonation to yield the neutral α-aminoalkyl radical TPrA^•^ (eq 3). This highly reactive species reduces the luminophore to [Ru­(bpy)_3_]^+^ (eq 4), that is ultimately promoted to the excited state following oxidation by TPrA^•+^ (eq 5).

Iteratively adjusting the rate constant of eq 3 in finite-element simulations aims to reproduce the TEL, which reflects the diffusion length of TPrA^•+^. Although this fitting approach yields a convenient estimate of the radical deprotonation rate (eq 6), the resulting constant is only an apparent parameter and lacks intrinsic physical meaning.

$$\mathrm{Radical}\;\mathrm{deprotonation}\;\mathrm{rate}=k_{H}\times [{\mathrm{TPrA}}^{•+}]$$ 6

In principle, if the reaction scheme and simulation framework perfectly reproduced the actual chemistry of the system, the fitted constant could converge toward the intrinsic rate constant. In practice, however, kinetic models rely on assumptions and simplifications: minor pathways may remain unidentified, reaction dynamics are typically simplified, and boundary conditions must be idealized to maintain acceptable computational costs. Mainly, the apparent deprotonation rate constant inherently includes the influence of several external factors, as predicted by eq 6: for instance, the pH governs the unprotonated amine concentration through acid–base equilibrium defined by its p*K*_a_ (eq 1), and the resulting coreactant concentration directly affects eq 2 that controls [TPrA^•+^] in eq 6. Likewise, the buffer concentration modulates proton-transfer dynamics and can further alter the apparent kinetics. Accordingly, the widely cited deprotonation constant of 3500 s^–1^ reflects the specific experimental and modeling conditions under which it was obtained, rather than the intrinsic reactivity of the radical cation.

Contrarily to experimental approaches, DFT in the framework of a hybrid cluster-continuum method, offers a means to estimate intrinsic kinetics at the molecular level. In DFT, a reaction pathway is represented as a trajectory on the molecular free-energy surface connecting reactants to products, with the kinetic barrier defined by the activation free energy (*ΔG* ^‡^) at the highest point along the minimum-energy path—the transition state.

For deprotonation of tertiary amine radical cations, this pathway corresponds to the transfer of a proton from the α-carbon adjacent to the nitrogen to a water molecule or to a small cluster of water molecules. Quantifying Δ*G* ^‡^ provides direct access, through transition state theory, to the intrinsic rate constant, thereby capturing the inherent molecular reactivity at infinite dilution, free from mass transport constraints, and independent of experimental variables. In this way, DFT complements experimental observations by supplying predictive, condition-free benchmarks for radical stability and reactivity, thereby helping disentangle fundamental kinetic factors from environmental influences. A detailed description of the computational approach is available in the Supporting Information (Figures S1–S5). All DFT-derived kinetic parameters discussed hereafter are summarized in Table 1, alongside the corresponding apparent constants reported in the literature. Mapping the free-energy profile of TPrA^•+^ deprotonation (Figure 2, blue path, Figures S1 and S2) identifies a Δ*G* ^‡^ of 12.8 kcal·mol^–1^ that, when fed into the Eyring eq (eq 7, see Supporting Information for kinetic details), results in a first-order rate constant of 3.1·10^3^ s^–1^.

$$k_{H}=\kappa \left(k_{B}\times T\right)/h\times \exp\left((-\Delta G^{‡}\right)/\mathrm{RT})$$ 7

**Figure 2:**
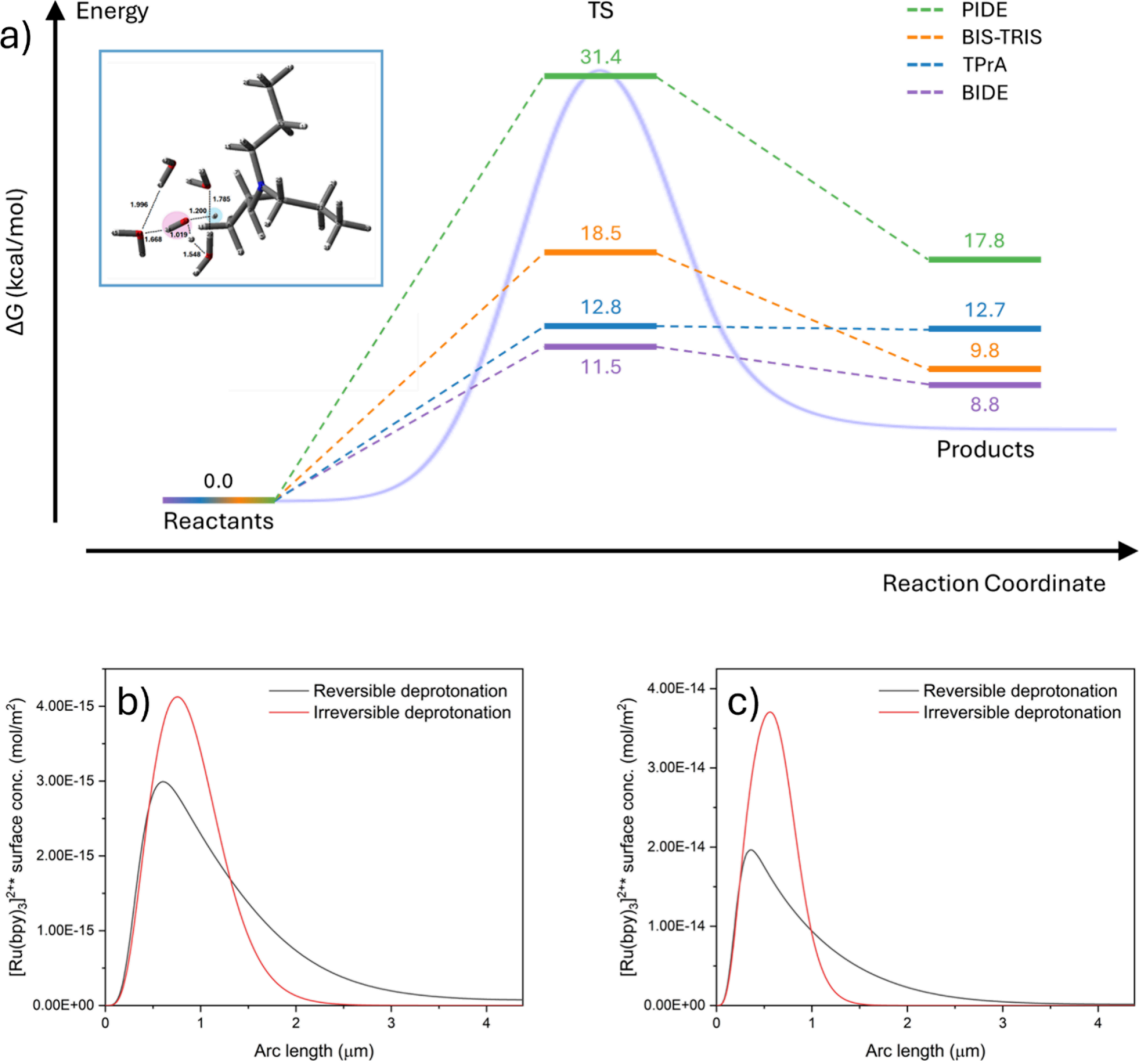
(a) M062X-D3-PCM/def2-TZVP-computed free energy profiles for the deprotonation of TPrA^•+^ (blue path), BIDE^•+^ (purple path), BIS-TRIS^•+^ (orange path), and PIDE^•+^ (green path) by explicit water molecules. The scheme represents the Gibbs free energies (kcal·mol^–1^, 300 K, 1 M standard state) of the reactant complexes (left), transition states (center), and product complexes (right). A hybrid cluster-continuum approach, including five explicit water molecules, was adopted. The blue curve in the background illustrates a representative reaction free energy pathway, including the activation barrier separating reactants and products. Inset: Optimized transition-state cluster including TPrA^•+^ and five water molecules, computed at the M062X-D3-PCM/def2-TZVP level. (b, c) Simulated concentration profiles of excited [Ru­(bpy)_3_]^2+^* on the bead surface in the presence of either (a) TPrA or (b) BIDE as coreactant. The deprotonation step was treated as both reversible (grey lines) and irreversible (red lines).

**Table 1:** Intrinsic Deprotonation Parameters (Δ*G* ^‡^ and *k*_H_) Obtained from DFT Calculations and Corresponding Apparent Rate Constants (*k*_apparent_) Reported in the Literature for the Investigated Coreactants, Enabling Direct Comparison between Intrinsic and Experimental Kinetics^a^

	ΔG^‡^ (kcal·mol^–1^)	k_H_ (s^–1^)	*k*_apparent_ (s^–1^)
TPrA	12.8	3.1·10^3^	3.5·10^3^
BIDE	11.5	2.6·10^4^	3.5·10^4^
BIS-TRIS	18.5	2.0·10^–1^	2.1·10^3^
PIDE	31.4	8.1·10^–11^	

a The apparent rate constants have been retrieved from References.^21,32^

The computed intrinsic deprotonation rate constants closely match the apparent value previously inferred from ECL imaging experiments. This agreement indicates that, for TPrA, the current mechanistic picture already captures the dominant reaction pathways and that eventual side processes play a negligible role. In other words, existing finite-element simulations provide a faithful representation of the underlying chemistry.

To experimentally assess how intrinsic deprotonation kinetics translate into light emission efficiency, ECLM was employed to image ECL generation from individual [Ru­(bpy)_3_]^2+^-functionalized microbeads. Details about ECL data processing are provided in the Supporting Information. In an inverted configuration (Figures 1 and S6), the focal plane under these conditions encompasses the whole TEL of the bead (see Supporting Information). As a result, integrated photon counts can be extracted as a quantitative measure of emission efficiency that include contributions from the ECL emitting layer (Figure S7). Single-bead ECLM measurements acquired using TPrA as the coreactant reveal intense emission centered on the microspheres surface (Figures 3 and S8a), consistent with its established role as a benchmark coreactant in bead-based ECL assays. These results validate single-bead ECL intensity as a robust experimental observable for correlating intrinsic radical kinetics with ECL efficiency.

**Figure 3:**
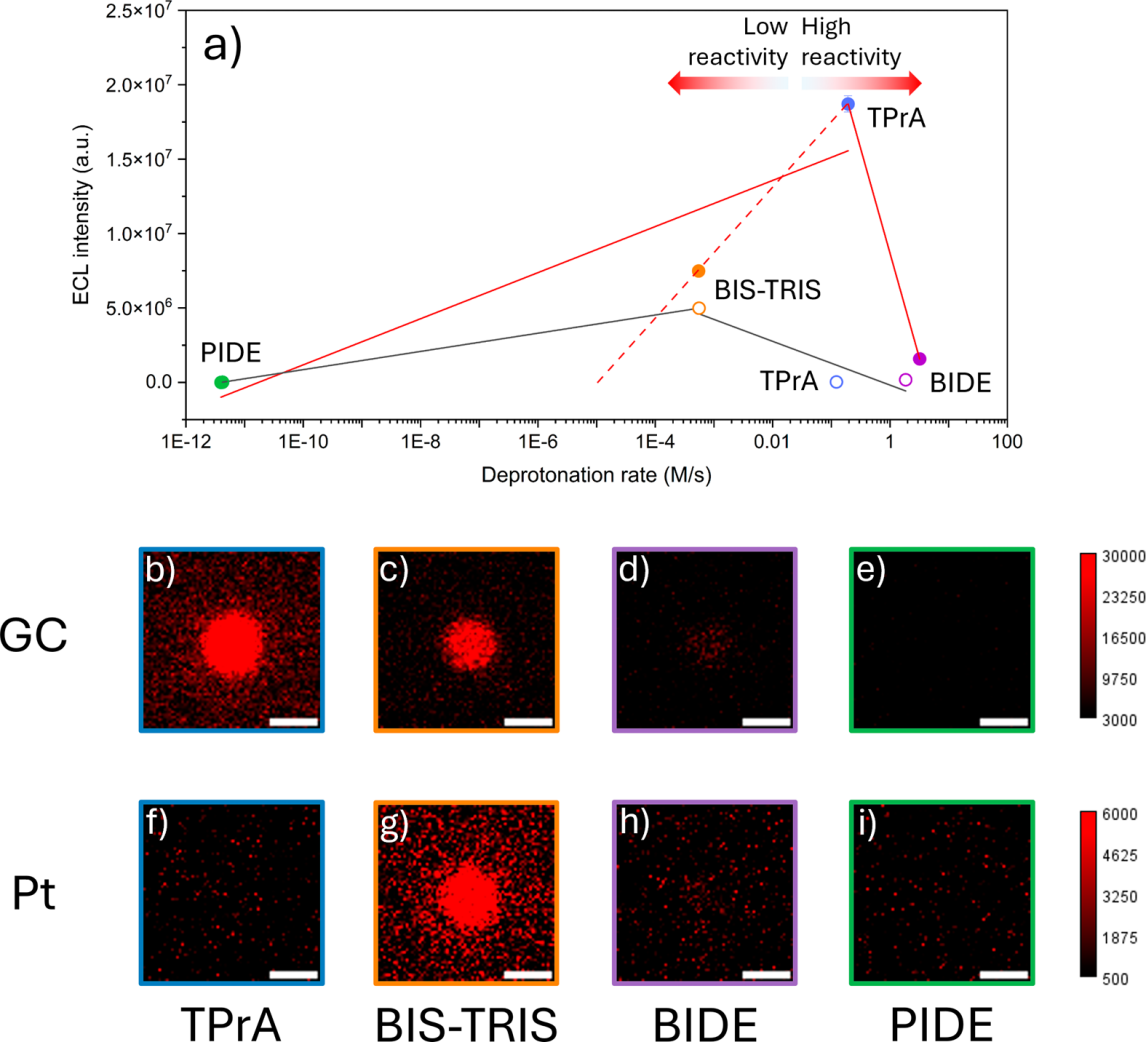
(a) Single-bead ECL intensity on GC (solid circles) and Pt (hollow circles) expressed as a function of the deprotonation rates of the corresponding radical cations. ECL intensities were determined from ECL images, by integrating the signal over a 21.5 μm^2^ region of interest (ROI) centered on the beads, subtracting the dark noise, and averaging the results over a minimum of seven beads (*n* ≥ 7). Error bars represent the standard error; when not visible, they are smaller than the symbol size. The semi-empirical deprotonation rates were determined by multiplying the DFT-derived rate constants by the simulated radical cation concentration at the electrode surface (Table S7). Solid lines represent linear fits of the two sides of the volcano plot on GC (red lines) and Pt (grey lines), while the dashed red line represents the linear fit between TPrA and BIS-TRIS on GC. These trend lines are included as a guide to visualize the ECL-reactivity relationship. (b–i) ECL images of [Ru­(bpy)_3_]^2+^-functionalized beads in 0.3 M PB with 0.1 M (b, f) TPrA, (c, g) BIS-TRIS, or (d, h) BIDE (pH 6.8) or in (e, i) 0.3 M acetate buffer with 0.1 M PIDE (pH 5). The images were captured on (b–e) GC and (h, i) Pt electrodes with an EM-CCD camera during a two-step chronoamperometry measurement: the ECL signal was recorded for 4 s (b–e) or 1 s (f–i) while holding the electrode at 1.7 V vs Ag/AgCl, after a 2 s step at open circuit potential. Magnification, ×100; objective numerical aperture, 0.8; gain, 5; sensitivity, 1200; contrast scale, 3000–30000 (b–e) and 500–6000 (h, i); scale bar, 3 μm.

The same DFT approach applied to BIS-TRIS reveals a *ΔG* ^‡^ of 18.5 kcal·mol^–1^ (Figure 2, orange path, Figure S3), and a corresponding rate constant of 2.03·10^–1^ s^–1^. In this case, the intrinsic deprotonation rate constant of BIS-TRIS^•+^ differs by several orders of magnitude from the apparent value previously reported in the literature. This discrepancy originates not from a failure of the deprotonation mechanism itself, which was verified via mass spectrometry,^32^ but most likely from the assumptions used to extract the apparent constant. Specifically, the apparent deprotonation rate of BIS-TRIS was determined by referencing it to TPrA, implicitly assuming identical effective concentrations. This approximation is problematic because of the substantial difference in their acidity: BIS-TRIS (p*K*_a_ = 6.46) exists predominantly in its unprotonated, electroactive form under neutral conditions, whereas TPrA is largely protonated. As a consequence, the effective concentration of electroactive BIS-TRIS is significantly higher than that of TPrA under identical nominal conditions. To support this interpretation, when the intrinsic deprotonation rate constant of BIS-TRIS is multiplied by its true radical cation concentration (eq 6), the resulting effective deprotonation rate becomes comparable to the apparent value obtained under the TPrA-referenced approximation (see Supporting Information).

A similar, although less pronounced, effect is observed for BIDE. Application of the same free-energy mapping approach to BIDE discloses a *ΔG* ^*‡*^ of 11.5 kcal·mol^–1^ (Figure 2, purple path, Figure S4), and a corresponding intrinsic deprotonation rate constant of 2.6·10^4^ s^–1^, which is smaller than the apparent value by approximately 1·10^4^ s^–1^. As in the case of BIS-TRIS, this deviation is related to differences between intrinsic and apparent rate constants arising from acid–base speciation effects. However, because the p*K*_a_ of BIDE (8.9) is closer to that of TPrA, the difference in effective electroactive concentrations is reduced, leading to a correspondingly smaller deviation from the literature value. This analysis demonstrates that the apparent discrepancy arises from differences in acid–base speciation rather than from fundamentally different radical reactivity, highlighting the limitations of apparent rate constants when comparing chemically dissimilar coreactants. Surprisingly, DFT calculations also reveal that the transition states for TPrA^•+^ and BIDE^•+^ deprotonation lie energetically close to the corresponding products (i.e., reducing α-aminoalkyl radical), showing a *ΔG* of 0.1 and 2.7 kcal·mol^–1^, respectively. Although the transition state and the product of TPrA appear essentially isoenergetic based on the calculations reported in Figure 2, this small free energy difference should be taken with caution. It primarily reflects close energetic proximity of these species, as documented by computations at various levels of theory that place *ΔG* within a narrow range of approximately 0.1–3 kcal/mol (see Supporting Information). Despite the reversibility of radical cations deprotonation has never been caught electrochemically,^26^ suggesting a fast and apparently irreversible process, DFT calculations provide computational support for earlier hypotheses based on pulse-radiolysis and ECL imaging experiments.^16,39^ These results indicate that proton transfer in tertiary amine radical cations can approach a dynamic equilibrium in proton-donating environments, characterized by its own p*K*_a_.^39–45^ Yet, typical ECL conditions tend to shift the system toward deprotonation, making the process appear irreversible, as also supported by finite element simulations (Figures 2b,c, S9, see Supporting Information for further discussion). Single-bead ECLM measurements were performed using BIDE and BIS-TRIS as coreactants under the same electrochemical and optical conditions employed for TPrA. In both cases, the single-bead ECL intensity is reduced compared to TPrA (Figures 3 and S8b,c). Nonetheless their opposing kinetic regimes, either less or more stable radicals compared to TPrA^•+^, hint that this behavior arises from fundamentally different mechanistic reasons. For BIDE, the intrinsically fast deprotonation of BIDE^•+^ leads to rapid decay of radical cations, limiting their diffusion length and resulting in a thinner ECL-emitting layer, as already stated in complementary experimental studies.^21,32^ The results with BIS-TRIS are more controversial. This molecule has been reported elsewhere as a more effective bead-based ECL coreactant than TPrA, in contrast to the results reported in the present work.^21,32,46^ Nevertheless, when Pt is used as the working electrode, BIS-TRIS triggers higher ECL intensity than TPrA (see Supporting Information), in agreement with previous reports and thereby reconciling this apparent discrepancy. The experimentally-independent deprotonation kinetics suggests that the formation rate of the reactive α-amino radical required to drive the ECL cascade is slowed down for BIS-TRIS. As a result, despite a thicker ECL emitting layer,^21,32^ the overall emission efficiency is reduced on the GC electrode.

Taken together, the contrasting behaviors of TPrA, BIDE, and BIS-TRIS indicate that bead-based ECL efficiency is not governed only by radical cation stability, but instead by a balance between radical lifetime and chemical reactivity. To rationalize these trends across a much broader kinetic spectrum, we propose PIDE (see Supporting Information for a comprehensive characterization, Figures S10 and S11), an aniline-derived tertiary amine that has not previously been explored as an ECL coreactant (Figure 1d). Contrarily to alkyl amines, the structural features encountered in PIDE are expected to stabilize the radical cation through aromatic spin delocalization while preserving sufficient aqueous solubility and suppressing deleterious radical–radical coupling. Consistently, deprotonation of PIDE^•+^ via DFT reveals a transition state lying at 31.4 kcal·mol^–1^ (Figure 2, green path, Figure S5), matched by a rate constant of 8.1·10^–11^ s^–1^. Such a small quantity firmly places this process in the slow-deprotonation regime. This compound therefore provides access to a previously unexplored region of radical stability in bead-based ECL systems and enables direct experimental interrogation of the long-lived limit of the kinetic landscape. Although forming an unusually long-lived radical cation, as predicted by DFT calculations, no detectable ECL emission was observed from individual [Ru­(bpy)_3_]^2+^-functionalized microbeads when PIDE was employed as the coreactant (Figures 3 and S8d).

To connect experimental observations into a unified kinetic framework, single-bead ECL emission was plotted as a function of the semi-empirical deprotonation rate (Figure 3a, eq 6). The semi-empirical nature arises from combining DFT-derived deprotonation rate constants (*k*_H_) with simulated radical cation concentrations based on experimental oxidation kinetics (see Supporting Information, Figure S12). In this representation, progressively slower deprotonation rates—corresponding to more stable radical cations—are found toward the left-hand side of the plot (low reactivity side in Figure 3a), whereas faster deprotonation and higher intrinsic reactivity lie toward the right (high reactivity side in Figure 3a). Thus, moving along the *x*-axis traces a gradual shift from short-lived, highly reactive intermediates to increasingly persistent radical cations. The resulting trend displays a non-monotonic dependence of emission intensity on deprotonation rate that reminds a volcano-type relationship: the ECL signal increases by slowing the deprotonation rate down to an optimal range, beyond which excessive radical stability quenches light emission. Despite BIDE being an attractive alternative to TPrA for reductive excitation ECL,^32,47,48^ it has been experimentally demonstrated to perform poorly in remote ECL.^21^ This behavior is associated with the small portion of the bead involved in the ECL process, where [Ru­(bpy)_3_]^2+^ labels lie within the very limited diffusion distance of BIDE^•+^. TPrA occupies the top of the plot, as its radical cation currently represents the ideal compromise between reactivity and stability. Moving beyond TPrA, on the descending side of the curve, BIS-TRIS triggers weaker, yet easily detectable, ECL emission. Consistently, further deceleration of the deprotonation rate, as observed for PIDE, shuts the ECL efficiency completely off. The reduced emission intensity with BIS-TRIS, followed by the absence of detectable signal with PIDE outline a clear descending pattern of ECL intensity upon decreasing the deprotonation rate, as the formation rate of the reactive α-amino radical is progressively more suppressed. In other words, the radical becomes so persistent that it ceases to act as a reactive intermediate, effectively halting the ECL process.

These findings reveal a fundamental trade-off in coreactant design: while greater radical cation stability extends half-life and diffusion range, excessive stabilization suppresses the reactivity necessary for light emission. Efficient ECL generation therefore requires a delicate balance between persistence and reactivity—radical cations must survive long enough to reach [Ru­(bpy)_3_]^2+^ labels distant from the electrode, yet remain sufficiently reactive to engage in charge-transfer steps. Linear fitting was applied separately to the two branches of the volcano plot (Figure 3a, solid red lines). However, the low-reactivity branch exhibits a markedly poor fit. This deviation arises from the loss of ECL emission observed with PIDE, which lies well beyond the range spanned by TPrA and BIS-TRIS. Rather than indicating a breakdown of the model, this behavior suggests the existence of an effective kinetic cutoff, below which the ECL process becomes inefficient and generation of the excited state collapses. Extrapolation of the low-reactivity branch (Figure 3a, dashed red line) indicates that ECL emission vanishes at deprotonation rates on the order of 3·10^–6^ M·s^–1^, implying that complete suppression of ECL occurs well before reaching the extreme stability regime represented by PIDE. On the high-reactivity side, instead, the weak ECL intensity already observed for BIDE indicates that the system is already approaching the upper kinetic limit. Notably, the two branches of the volcano plot exhibit markedly different slopes, with the high-reactivity branch being approximately 3.2 times steeper than the low-reactivity branch. This asymmetry indicates that ECL efficiency responds differently to variations in deprotonation kinetics across the two sides of the volcano plot. The emission intensity is considerably more sensitive to kinetic variations in the fast-deprotonation regime, where TEL is severely constrained, as variations in radical decay translate into large changes in the number of addressable luminophores. On the other hand, in the slow-deprotonation regime, where the emitting layer already extends over most of the bead surface, the increased number of active [Ru­(bpy)_3_]^2+^ labels partially compensates for slower deprotonation, attenuating the impact of further kinetic stabilization on emission intensity. To evaluate whether the observed volcano-type dependence reflects intrinsic coreactant kinetics rather than electrodic effects, the same analysis was performed using Pt as the working electrode (Figures 3a and S13). In this case, the relative ECL intensities differ from those obtained on GC, with BIS-TRIS displaying the highest signal, while TPrA and PIDE remain essentially inactive and BIDE yields only marginal emission above background. This reorganization is consistent with Tafel analysis, which indicates that the oxidation kinetics of BIDE and, more markedly, TPrA are slowed down on a more hydrophilic electrode, whereas BIS-TRIS exhibits comparatively weaker electrode dependence. Despite these variations in oxidation behavior, the non-monotonic dependence of ECL intensity on deprotonation rate is preserved, demonstrating that the volcano profile primarily reflects intrinsic radical-cation kinetics rather than electrodic effects.

## 4 Conclusions

Correlating the ECL intensity from single labeled beads with the semi-empirical decomposition rate of tertiary amines radical cations reveals an unexpected nonlinear, volcano-type behavior. DFT simulations disentangle deprotonation kinetics from experimental effects, transforming apparent rate constants into molecular descriptors that directly reflect structural properties. This approach combining experimental and computation chemistry explicitly establishes, for the first time, the concept of an optimal radical half-life window for bead-based ECL systems. Within this range, radicals are sufficiently long-lived to reach relatively distant luminophores yet remain reactive enough to sustain ECL generation. By providing access to intrinsic rate constants independent of experimental constraints, DFT complements empirical approaches and redefines the paradigm of ECL coreactant development from the pursuit of maximum radical stability to the identification of a balanced stability–reactivity regime. In this context, quantum-chemical evaluation of deprotonation barriers emerges as a powerful tool to pre-screen molecular candidates within the newly defined optimal lifetime window, bridging mechanistic understanding with practical molecular innovation.

## Supplementary Material



## Data Availability

The data that support the findings of this study are openly available in AMS Acta at https://amsacta.unibo.it/id/eprint/8882.

## Notes

Chemicals, instrumentation, experimental and computational details; ECL image processing methods; COMSOL simulation model; single-bead ECL images; electrochemical and computational characterization of PIDE; and Tafel analysis (PDF)

## References

[1] Lewis, T. H. ; Wan, R. ; Leininger, W. R. ; Zhang, B. Electrochemical Imaging. ACS In Focus; American Chemical Society, 2024.

[2] Knežević S., Totoricaguena-Gorriño J., Gajjala R. K. R., Hermenegildo B., Ruiz-Rubio L., Vilas-Vilela J. L., Lanceros-Méndez S., Sojic N., Del Campo F. J. (2024). Enhanced Electrochemiluminescence
at the Gas/Liquid Interface of Bubbles Propelled into Solution. J. Am. Chem. Soc..

[3] Han D., Sojic N., Jiang D. (2025). Spatial Profiling
of Multiple Enzymatic
Activities at Single Tissue Sections via Fenton-Promoted Electrochemiluminescence. J. Am. Chem. Soc..

[4] Fracassa A., Ferrari G., Balli M. V., Rimoldi I., Facchetti G., Arnal L., Marconi A., Calvaresi M., Prodi L., De Cola L., Valenti G. (2025). Stimuli-Responsive
Luminophore Drives Mechanism Switch for Highly Efficient Electrochemiluminescence
Immunosensing. J. Am. Chem. Soc..

[5] Xiang Z., Wei C., Zhu S., Liu S., Zhang R., Tong X., Wang Z., Deng J., Zhang X., Zhong L., Sun R., Hou Y., Jiang C., Zhang Y., Luo Y., Zang G. (2026). From Detection to Mechanistic Insight: Electrochemiluminescence as
a Universal Platform for Reaction Kinetics. Biosens. Bioelectron..

[6] Layman B. R., Hill M. L., Dick J. E. (2026). Radical Electroprecipitation
Prolongs
“Electro”Chemiluminescence of the Tris­(2,2’-Bipyridyl)­Ruthenium­(II)
and Tri-n-Propylamine System by a Millionfold. J. Am. Chem. Soc..

[7] Sojic N., Knežević S., Han D., Liu B., Jiang D. (2024). Electrochemiluminescence Microscopy. Angew.
Chemi., Int. Ed..

[8] Xing Z., Lu X., Zhang Z., Zhao Y., Cao Y., Zhou Y., Zhu J. J. (2025). Electrochemiluminescence Microscopy in Nano-Electrochemistry
Research: Unraveling the Underlying Principles, Tracing the Evolutionary
Developments, and Charting the Prospective Trajectories. Adv. Funct. Mater..

[9] Rong Y., Lin C., Qi M., Fu W., Su B. (2026). Recent Advances in
Electrochemiluminescence Imaging Applications. Chem. Biomed. Imaging.

[10] Faatz, E. ; Finke, A. ; Josel, H. P. ; Prencipe, G. ; Quint, S. ; Windfuhr, M. Chapter 15: Automated Immunoassays for the Detection of Biomarkers in Body Fluids. In Analytical Electrogenerated Chemiluminescence; Royal Society of Chemistry, 2019; pp 443–470.

[11] Barhoum A., Altintas Z., Devi K. S. S., Forster R. J. (2023). Electrochemiluminescence
Biosensors for Detection of Cancer Biomarkers in Biofluids: Principles,
Opportunities, and Challenges. Nano Today.

[12] Yu J., Stankovic D., Vidic J., Sojic N. (2024). Recent Advances in
Electrochemiluminescence Immunosensing. Sens.
Diagn..

[13] Giagu G., Fracassa A., Fiorani A., Villani E., Paolucci F., Valenti G., Zanut A. (2024). From Theory
to Practice: Understanding
the Challenges in the Implementation of Electrogenerated Chemiluminescence
for Analytical Applications. Microchim. Acta.

[14] Sentic M., Milutinovic M., Kanoufi F., Manojlovic D., Arbault S., Sojic N. (2014). Mapping Electrogenerated Chemiluminescence
Reactivity in Space: Mechanistic Insight into Model Systems Used in
Immunoassays. Chem. Sci..

[15] Zanut A., Fiorani A., Canola S., Saito T., Ziebart N., Rapino S., Rebeccani S., Barbon A., Irie T., Josel H. P., Negri F., Marcaccio M., Windfuhr M., Imai K., Valenti G., Paolucci F. (2020). Insights into
the Mechanism of Coreactant Electrochemiluminescence Facilitating
Enhanced Bioanalytical Performance. Nat. Commun..

[16] Fiorani A., Han D., Jiang D., Fang D., Paolucci F., Sojic N., Valenti G. (2020). Spatially Resolved
Electrochemiluminescence through
a Chemical Lens. Chem. Sci..

[17] Dutta P., Han D., Goudeau B., Jiang D., Fang D., Sojic N. (2020). Reactivity
Mapping of Luminescence in Space: Insights into Heterogeneous Electrochemiluminescence
Bioassays. Biosens. Bioelectron..

[18] Han D., Fang D., Valenti G., Paolucci F., Kanoufi F., Jiang D., Sojic N. (2023). Dynamic Mapping
of Electrochemiluminescence
Reactivity in Space: Application to Bead-Based Assays. Anal. Chem..

[19] Han D., Jiang D., Valenti G., Paolucci F., Kanoufi F., Chaumet P. C., Fang D., Sojic N. (2023). Optics Determines the
Electrochemiluminescence Signal of Bead-Based Immunoassays. ACS Sensors.

[20] Feng Y., Zhou W., Wang X., Zhang J., Zou M., Zhang C., Qi H. (2023). Imaging and
Simulation of Ruthenium
Derivative Coating Microbeads at the Opaque Electrode with Electrogenerated
Chemiluminescence. Chem. Biomed. Imaging.

[21] Feng Y., Wang C., Zhou W., Yang X., Paolucci F., Valenti G., Qi H. (2025). Tomography Electrogenerated Chemiluminescence
Imaging from Magnetic Microbeads. Small.

[22] Ding J., Wang Y., Arias-Aranda L. R., Chaumet P. C., Sojic N., Su B. (2025). Transparent Single-Layer
Graphene Electrodes for Deciphering and
Enhancing Microbead-Based Electrochemiluminescence Immunoassay. Small.

[23] Kerr E., Knezevic S., Francis P. S., Hogan C. F., Valenti G., Paolucci F., Kanoufi F., Sojic N. (2023). Electrochemiluminescence
Amplification in Bead-Based Assays Induced by a Freely Diffusing Iridium­(III)
Complex. ACS Sensors.

[24] Fracassa A., Santo C. I., Kerr E., Knežević S., Hayne D. J., Francis P. S., Kanoufi F., Sojic N., Paolucci F., Valenti G. (2024). Redox-Mediated
Electrochemiluminescence
Enhancement for Bead-Based Immunoassay. Chem.
Sci..

[25] Kerr E., Doeven E. H., Francis P. S. (2022). Recent Advances in Mechanistic Understanding
and Analytical Methodologies of the Electrochemiluminescence of Tris­(2,2′-Bipyridine)­Ruthenium­(II)
and Tri-n-Propylamine. Curr. Opin. Electrochem..

[26] Miao W., Choi J. P., Bard A. J. (2002). Electrogenerated
Chemiluminescence
69: The Tris­(2,2′-Bipyridine)­Ruthenium­(II), (Ru­(Bpy)­32+)/Tri-n-Propylamine
(TPrA) System Revisited - A New Route Involving TPrA.+ Cation Radicals. J. Am. Chem. Soc..

[27] Daviddi E., Oleinick A., Svir I., Valenti G., Paolucci F., Amatore C. (2017). Theory and Simulation
for Optimising Electrogenerated
Chemiluminescence from Tris­(2,2′-Bipyridine)-Ruthenium­(II)-Doped
Silica Nanoparticles and Tripropylamine. ChemElectroChem..

[28] Guo W., Zhou P., Sun L., Ding H., Su B. (2021). Microtube
Electrodes for Imaging the Electrochemiluminescence Layer and Deciphering
the Reaction Mechanism. Angew. Chemie - Int.
Ed..

[29] Fu W., Zhou P., Guo W., Su B. (2022). Imaging Electrochemiluminescence
Layer to Dissect Concentration-Dependent Light Intensity for Accurate
Quantitative Analysis. Adv. Sens. Energy Mater..

[30] Mariani C., Fracassa A., Pastore P., Bogialli S., Paolucci F., Valenti G., Zanut A. (2025). Singling Out
the Electrochemiluminescence
Profile in Microelectrode Arrays. Chem. Biomed.
Imaging.

[31] Wang Y., Guo W., Yang Q., Su B. (2020). Electrochemiluminescence Self-Interference
Spectroscopy with Vertical Nanoscale Resolution. J. Am. Chem. Soc..

[32] Wang Y., Ding J., Zhou P., Liu J., Qiao Z., Yu K., Jiang J., Su B. (2023). Electrochemiluminescence
Distance
and Reactivity of Coreactants Determine the Sensitivity of Bead-Based
Immunoassays. Angew. Chem., Int. Ed..

[33] Yang X., Hang J., Qu W., Wang Y., Wang L., Zhou P., Ding H., Su B., Lei J., Guo W., Dai Z. (2023). Gold Microbeads Enabled
Proximity Electrochemiluminescence
for Highly Sensitive and Size-Encoded Multiplex Immunoassays. J. Am. Chem. Soc..

[34] Xu H., Luo R., Lv H., Liu T., Liao Q., Wang Y., Zhong Z., Wu X., Lei J., Xi K. (2025). Deciphering
a Volcano-Shaped Relationship between Radical Stability and Reticular
Electrochemiluminescence. Nat. Commun..

[35] Zhang X., Jie J., Song D., Su H. (2020). Deprotonation
of Guanine Radical
Cation G•+Mediated by the Protonated Water Cluster. J. Phys. Chem. A.

[36] Kanoufi F., Zu Y., Bard A. J. (2001). Homogeneous
Oxidation of Trialkylamines by Metal Complexes
and Its Impact on Electrogenerated Chemiluminescence in the Trialkylamine/Ru­(Bpy)­32+
System. J. Phys. Chem. B.

[37] Wightman R. M., Forry S. P., Maus R., Badocco D., Pastore P. (2004). Rate-Determining
Step in the Electrogenerated Chemiluminescence from Tertiary Amines
with Tris­(2,2‘-Bipyridyl)­Ruthenium­(II). J. Phys. Chem. B.

[38] Pastore P., Badocco D., Zanon F. (2006). Influence
of Nature, Concentration
and PH of Buffer Acid-Base System on Rate Determining Step of the
Electrochemiluminescence of Ru­(Bpy)­32+ with Tertiary Aliphatic Amines. Electrochim. Acta.

[39] Das S., von Sonntag C. (1986). The Oxidation
of Trimethylamine by OH Radicals in Aqueous
Solution, as Studied by Pulse Radiolysis, ESR, and Product Analysis.
The Reactions of the Alkylamine Radical Cation, the Aminoalkyl Radical,
and the Protonated Aminoalkyl Radical. Zeitschrift
fur Naturforsch. - Sect. B J. Chem. Sci..

[40] de
Nicholas A. M. P., Arnold D. R. (1982). Thermochemical Parameters for Organic
Radicals and Radical Ions. Part 1. The Estimation of the p K a of
Radical Cations Based on Thermochemical Calculations. Can. J. Chem..

[41] Nelsen S. F., Ippoliti J. T. (1986). On the Deprotonation of Trialkylamine Cation Radicals
by Amines. J. Am. Chem. Soc..

[42] Dinnocenzo J. P., Banach T. E. (1989). Deprotonation of
Tertiary Amine Cation Radicals. A
Direct Experimental Approach. J. Am. Chem. Soc..

[43] Okazaki O., Guengerich F. P. (1993). Evidence for Specific Base Catalysis in N-Dealkylation
Reactions Catalyzed by Cytochrome P450 and Chloroperoxidase: Differences
in Rates of Deprotonation of Aminium Radicals as an Explanation for
High Kinetic Hydrogen Isotope Effects Observed with Peroxid. J. Biol. Chem..

[44] Dombrowski G. W., Dinnocenzo J. P., Zielinski P. A., Farid S., Wosinska Z. M., Gould I. R. (2005). Efficient
Unimolecular Deprotonation of Aniline Radical
Cations. J. Org. Chem..

[45] Yu A., Liu Y., Li Z., Cheng J. P. (2007). Computation of PKa Values of Substituted
Aniline Radical Cations in Dimethylsulfoxide Solution. J. Phys. Chem. A.

[46] Fu W., Wang X., Ying X., Sun T., Wang Y., Wang J., Su B. (2024). Electrochemiluminescence
Lateral
Flow Immunoassay Using Ruthenium­(II) Complex-Loaded Dendritic Mesoporous
Silica Nanospheres for Highly Sensitive and Quantitative Detection
of SARS-CoV-2 Nucleocapsid Protein. Adv. Funct.
Mater..

[47] Han S., Niu W., Li H., Hu L., Yuan Y., Xu G. (2010). Effect of
Hydroxyl and Amino Groups on Electrochemiluminescence Activity of
Tertiary Amines at Low Tris­(2,2′-Bipyridyl)­Ruthenium­(II) Concentrations. Talanta.

[48] Francis P. S., Knežević S., Hogan C. F., Sojic N. (2025). Terminology
of Electrochemiluminescence Reaction Mechanisms. ACS Electrochem..

