# Supplementary material for: Rationalized Volcano Plot in Heterogeneous Electrochemiluminescence
Source: ACS Electrochem. 2026 Apr 6;2(5):1324–32. doi: 10.1021/acselectrochem.6c00072 (PMC13159797; doi:10.1021/acselectrochem.6c00072)
Supplement: Supplementary file 1 [file ec6c00072_si_001.pdf]

Supporting Information

Rationalized Volcano Plot in Heterogeneous Electrochemiluminescence

Alessandro Fracassa,[‡] Michele Orza,[‡] Chiara Mariani, Claudio Ignazio Santo, Claudia Martinez Asenjo, Elisa D'Arrigo, Neso Sojic, Francesco Paolucci, Fabrizia Negri,* Giovanni Valenti*

Affiliations

[a] Department of Chemistry "Giacomo Ciamician", Alma Mater Studiorum – University of Bologna, 40129 Bologna, Italy

[b] Center for Chemical Catalysis – C3, Alma Mater Studiorum – University of Bologna, 40129 Bologna, Italy

[c] University of Bordeaux, CNRS UMR 5255, Bordeaux INP, ENSMAC, Pessac 33607, France

[d] CNR-ICMATE, Corso Stati Uniti 4, 35127 Padova, Italy

*Corresponding authors: fabrizia.negri@unibo.it; g.valenti@unibo.it

Contents

Chemicals ... **2**

Computational details ... **2**

DFT $TPrA^{•+}$ deprotonation pathway ... 3

DFT BIS-$TRIS^{•+}$ deprotonation pathway ... 5

DFT $BIDE^{•+}$ deprotonation pathway ... 6

DFT $PIDE^{•+}$ deprotonation pathway ... 7

Electrochemiluminescence ... **7**

ECL microscopy ... 7

Data elaboration: Integration of the ECL signal ... 8

Depth of field ... 9

ECL imaging on GC electrode ... 10

COMSOL simulations ... **11**

Simulated $[Ru(bpy)_3]^{2+*}$ profiles ... 15

PIDE characterization ... **16**

Tafel analysis and determination of electron-transfer kinetics ... **18**

ECL imaging on Pt electrode ... 20

References ... **21**

Chemicals

Bis(2,2′-bipyridine)-4′-methyl-4-carboxybipyridine-ruthenium N-succinimidyl ester-bis(hexafluorophosphate) ($[Ru(bpy)_2(mcbpy\text{-}O\text{-}Su\text{-}ester)](PF_6)_2$, MW = 1014.66 g·mol^{-1}), Tri-*n*-propylamine (TPrA, MW = 143.27 g·mol^{-1}, ≥98%), N-Butyldiethanolamine (BIDE, MW = 161.24 g·mol^{-1}, ≥98.6%), BIS-TRIS (MW = 209.24 g·mol^{-1}, BioUltra, ≥99.0%), N,N-Bis(2-hydroxyethyl)-p-toluidine (PIDE, MW = 195.26 g·mol^{-1}, ≥97.0%), sodium phosphate monobasic dihydrate ($NaH_2PO_4{\cdot}2H_2O$, MW = 156.01 g·mol^{-1}, ≥99%), sodium phosphate dibasic (Na_2HPO_4, MW = 141.96 g·mol^{-1}, ≥99.5%), phosphoric acid (H_3PO_4, MW = 98.00 g·mol^{-1}, ≥85%), and dimethyl sulfoxide (DMSO, $(CH_3)_2SO$, MW = 78.13 g·mol^{-1}, ≥99.7%) were purchased from Sigma-Aldrich.

Amine-coated 2.8 μm polystyrene beads (Dynabeads™ M-270 Amine) were purchased from Thermo Fisher Scientific Inc.

Computational details

All quantum chemical calculations were performed using the Gaussian 16 and ORCA 6.0.1 software packages.[1,2] An extensive conformational search was initially performed on each investigated tertiary amine with the GOAT (global geometry optimization and ensemble generator) algorithm as implemented in ORCA, employing the GFN2-xTB Hamiltonian[3] in combination with the ALPB implicit solvation model to efficiently sample the accessible conformational space under solvated conditions.[4] The resulting conformers were ranked according to their relative energies, and the 50 lowest-energy structures were selected and reoptimized at the DFT level, using the M06-2X functional with the def2-SVP basis set. The M062X functional[5] was chosen since it has been shown to well describe non-covalent interactions and reaction energetics. Additionally, to consider long-range electron correlation, Grimme's D3 dispersion correction was included.[6,7] Solvent effects were accounted for through an implicit solvation model based on the Polarizable Continuum Model (PCM).[8,9] The final DFT level of theory is therefore labelled M062X-D3-PCM. Harmonic vibrational frequency calculations were performed to confirm all stationary points as true minima on the potential energy surface.

Many theoretical and experimental investigations show that molecular mechanism behind proton transfer involves a water-assisted proton transfer with one or more explicit water molecules acting as the proton acceptor.[10] Thus, a hybrid cluster continuum approach[11] was employed, in which clusters formed by the tertiary amine radical cation and explicit water molecules (introduced to account for specific solute-solvent interactions relevant to the proton-transfer mechanism) are immersed in a dielectric continuum. Up to five explicit water molecules were considered and the corresponding complexes were reoptimized at DFT level (M062X-D3-PCM/def2-SVP). Transition-state structures were determined by relaxed potential energy surface scans along the proton-transfer coordinate and fully optimized at the same level of theory. Vibrational frequency analyses confirmed the transition state nature by the presence of a single imaginary frequency associated with the reaction coordinate. Intrinsic reaction coordinate (IRC) calculations were performed to verify the connectivity between transition states and the corresponding reactant and product minima, along the deprotonation pathway. Thermal corrections were evaluated at 300 K, and the Gibbs free energy of

activation ($\Delta G^{\ddagger}$) was obtained as the free-energy difference between the transition state and the solvated reactant complex (see also M062X-D3-PCM/def2-SVP data in Table S1 and in Figure S1). DLPNO-CCSD(T) calculations were also carried out at the optimized geometries in the case of TPrA (see Table S2). To improve the accuracy of the calculated reaction profiles, all stationary points determined at M062X-D3-PCM/def2-SVP level were subsequently refined by geometry optimization at M062X-D3-PCM/def2-TZVP level of theory. Harmonic vibrational frequency calculations performed at the same level of theory confirmed the nature of the optimized minima and transition states. The M062X-D3-PCM/def2-TZVP computed Gibbs free energy profiles are schematically represented in Figure S2-S5 (see also Table S1 for a comparison between calculated activation free energies ($\Delta G^{\ddagger}$) using the def2-SVP and def2-TZVP basis sets).

Table S1. Gibbs free energy barriers ($\Delta G^{\ddagger}$, kcal·mol^{-1}) for the deprotonation of the investigated tertiary amine radical cations.

Amine	$\Delta G^{\ddagger}$ (kcal/mol)[a]	$\Delta G^{\ddagger}$ (kcal/mol)[b]
TPrA	10.1	12.8
BIS-TRIS	18.0	18.5
BIDE	14.3	11.5
PIDE	34.4	31.4

[a]From M062X-D3-PCM/def2-SVP calculations. [b]From M062X-D3-PCM/def2-TZVP calculations.

Rate constants for the α-proton transfer of the amine radical cations were estimated using Transition State Theory (TST) based on density functional theory (DFT) calculations. Because the proton-transfer process occurs within a pre-formed, solvated reactive complex, the calculated $\Delta G^{\ddagger}$ corresponds to a unimolecular elementary step (intramolecular proton transfer within a solvent cage).

The corresponding rate constants k_H were computed using the Eyring equation:

$$k_H = \kappa \frac{k_B T}{h} exp\left(\frac{-\Delta G^{\ddagger}}{RT}\right)$$

where k_B is the Boltzmann constant, h is Planck constant, R is the ideal gas constant, and T is the temperature (300 K). The transmission coefficient κ was set to unity, i.e., quantum tunneling corrections were neglected. The resulting rate constant has units of s^{-1} and represents a pseudo–first-order decay constant for the deprotonation of solvated coreactant radical cations in water.

Notably, these calculated rate constants represent intrinsic chemical kinetics for the proton-transfer step and do not account for diffusion, electrochemical generation, or mass-transport effects, which were treated separately in the finite-element simulations.

DFT TPrA$^{\bullet+}$ deprotonation pathway

For TPrA$^{\bullet+}$ deprotonation, the ΔG between the transition state and the corresponding product was found in the order of a few kcal·mol^{-1} at different levels of theory (see Table S2), namely in the range 0.1-3 kcal·mol^{-1}. These values, once converted into rate constants, result in diffusion-limited reaction rates, consistently with shallow barriers along the protonation coordinate.

Table S2. Gibbs free energy differences (kcal·mol^{-1}) between TS and P computed for TPrA radical cation at different levels of theory.

Level of theory	ΔG(TS-P) (kcal/mol)
M062X-D3-PCM/def2-SVP[a]	2.1
DLPNO-CCSD(T) @ M062X-D3-PCM/def2-SVP geometry[a]	2.8
M062X-D3-PCM/def2-TZVP[a]	0.1
DLPNO @ M062X-D3-PCM/def2-TZVP geometry[a]	0.4
M062X-D3-PCM/def2-SVP[b]	3.0

[a]Including five explicit water molecules. [b]Including six explicit water molecules.

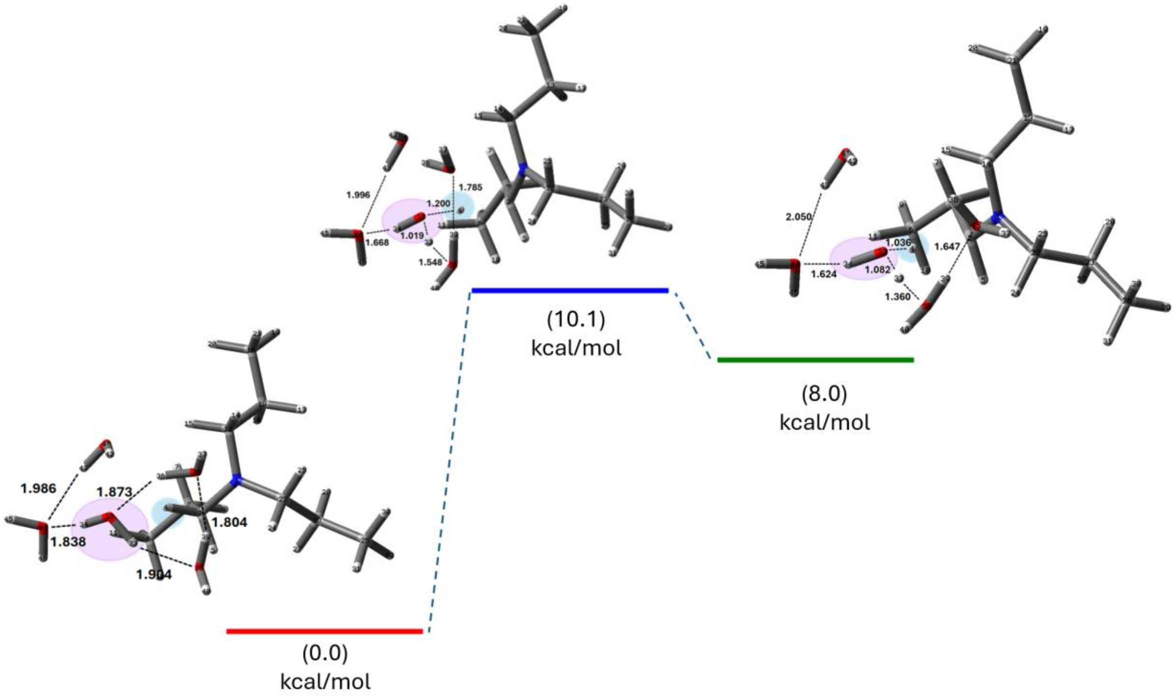

Figure S1. Reactant, transition state, and product structures for the TPrA$^{•+}$ deprotonation pathway along with the corresponding Gibbs free energy differences (kcal·mol^{-1}) relative to the reactant for both the transition state and the product. From M062X-D3-PCM/def2-SVP calculations including five explicit water molecules. The main water molecule involved in the reaction is highlighted in pink, while the transferred proton is highlighted in light blue.

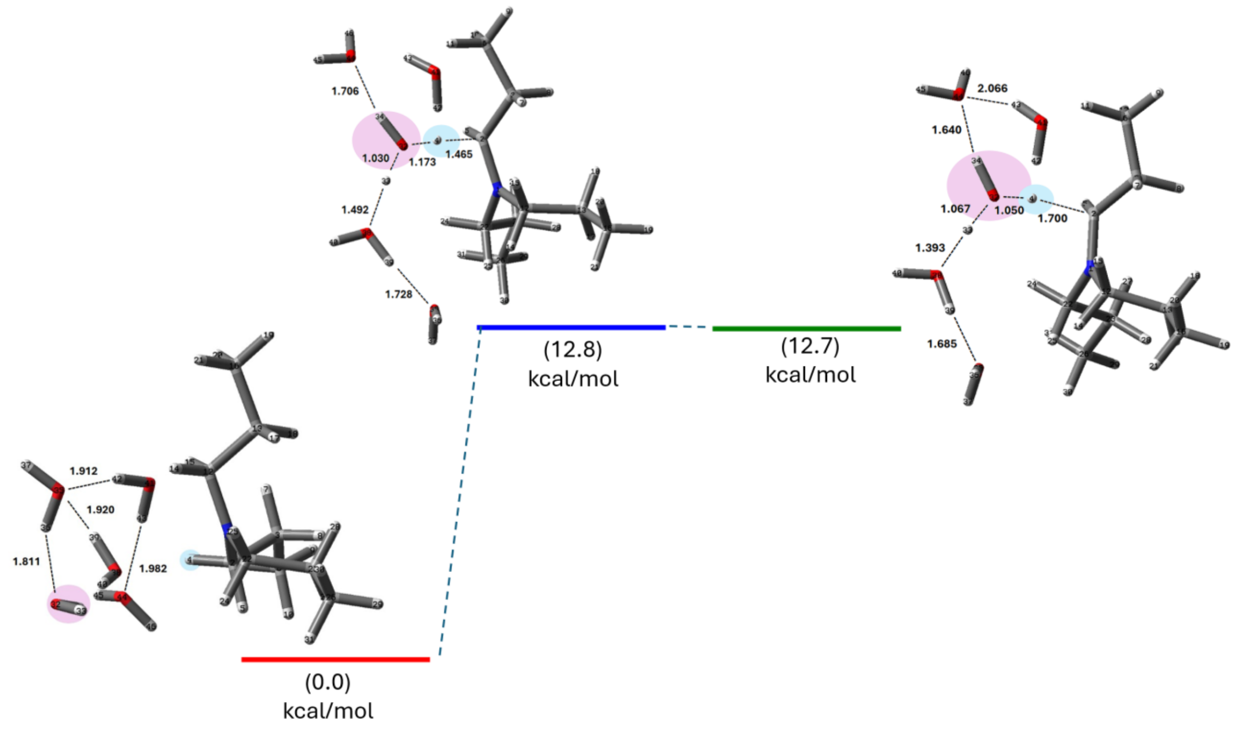

Figure S2. Reactant, transition state, and product structures for the TPrA$^{•+}$ deprotonation pathway along with the corresponding Gibbs free energy differences (kcal·mol^{-1}) relative to the reactant for both the transition state and the product. From M062X-D3-PCM/def2-TZVP calculations including five explicit water molecules. The main water molecule involved in the reaction is highlighted in pink, while the transferred proton is highlighted in light blue.

DFT BIS-TRIS$^{•+}$ deprotonation pathway

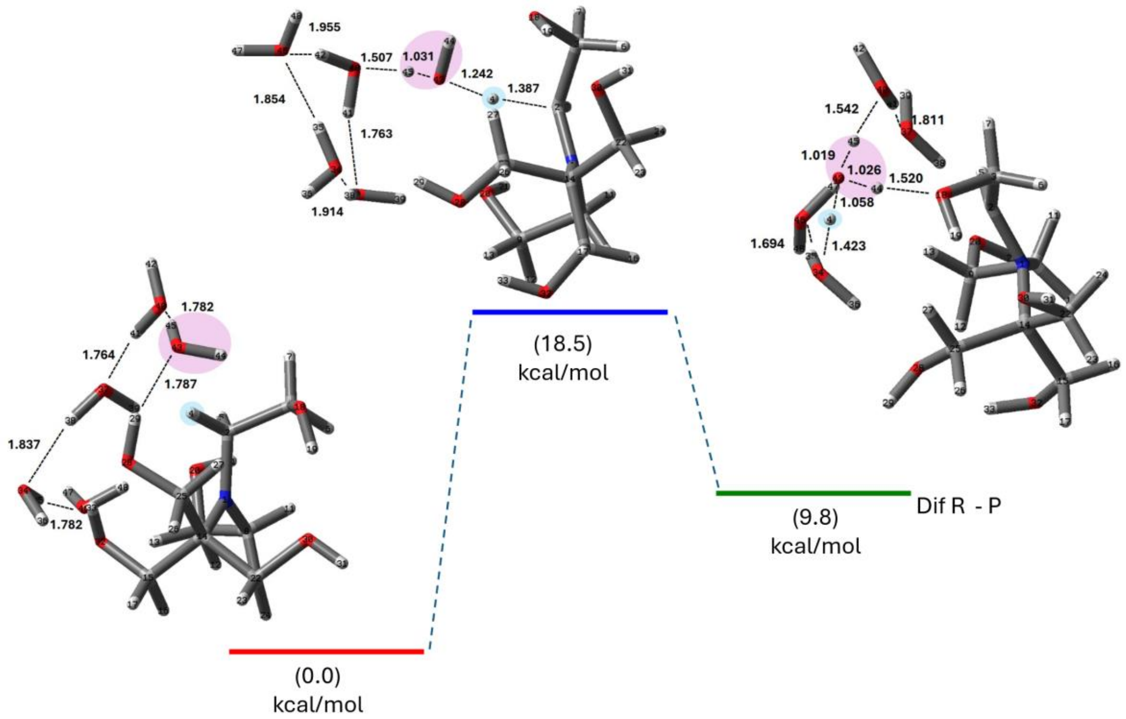

Figure S3. Reactant, transition state, and product structures for the BIS-TRIS$^{•+}$ deprotonation pathway along with the corresponding Gibbs free energy differences (kcal·mol^{-1}) relative to the reactant for both the transition state and the product. From M062X-D3-PCM/def2-TZVP calculations including five explicit water molecules. The main water molecule involved in the reaction is highlighted in pink, while the transferred proton is highlighted in light blue.

Under the assumption of identical oxidation kinetics (vide infra for k^0 determination), the simulated concentration of radical cation (vide infra for finite element modeling), BIS-TRIS$^{\bullet+}$, depends on the coreactant pK_a that governs the concentration of electroactive amine and on the deprotonation kinetics. In this context, the effective and apparent deprotonation rates can be estimated through Eq. 6. The calculated reaction rates of $1.4{\cdot}10^{-3}$ and $0.55{\cdot}10^{-3}$ $M{\cdot}s^{-1}$ for the parametrized and the intrinsic cases, respectively, indicate that although the intrinsic deprotonation constant of BIS-TRIS$^{\bullet+}$ is significantly slower than initially predicted, the high concentration of electroactive species compensates for this limitation, sustaining an overall comparable reaction rate.

DFT BIDE$^{\bullet+}$ deprotonation pathway

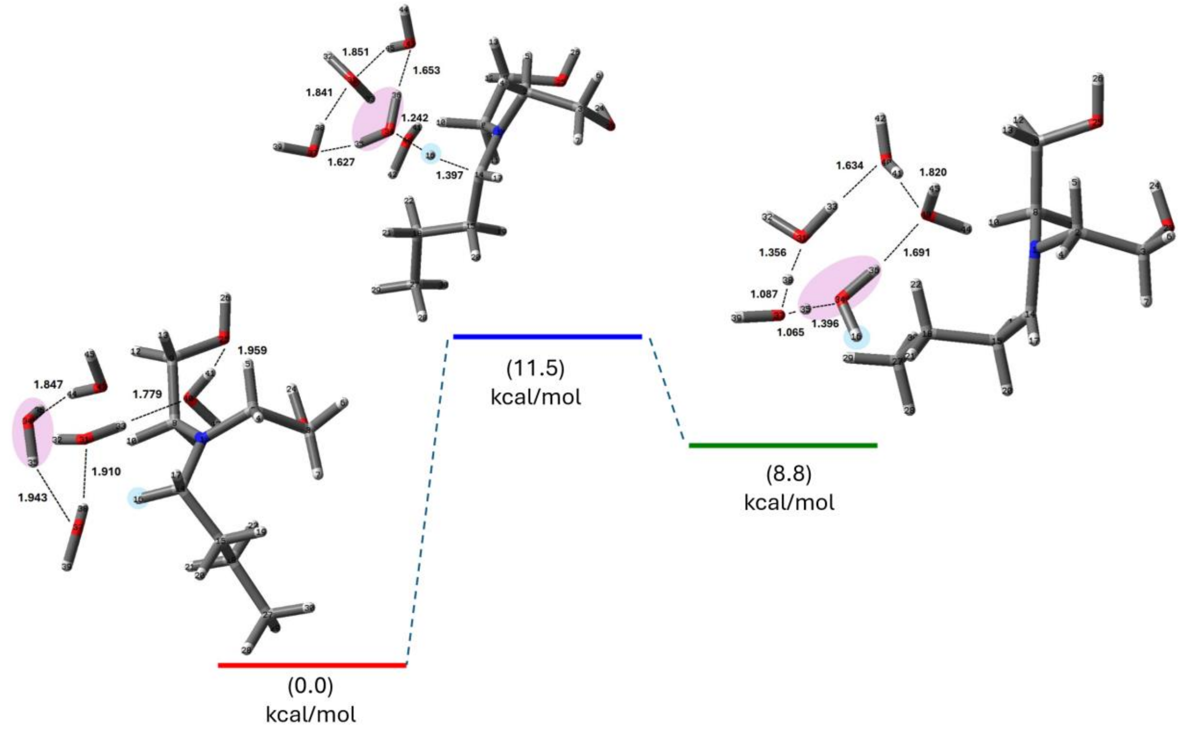

Figure S4. Reactant, transition state, and product structures for the BIDE$^{\bullet+}$ deprotonation pathway along with the corresponding Gibbs free energy differences ($kcal{\cdot}mol^{-1}$) relative to the reactant for both the transition state and the product. From M062X-D3-PCM/def2-TZVP calculations including five explicit water molecules. The main water molecule involved in the reaction is highlighted in pink, while the transferred proton is highlighted in light blue.

DFT PIDE$^{•+}$ deprotonation pathway

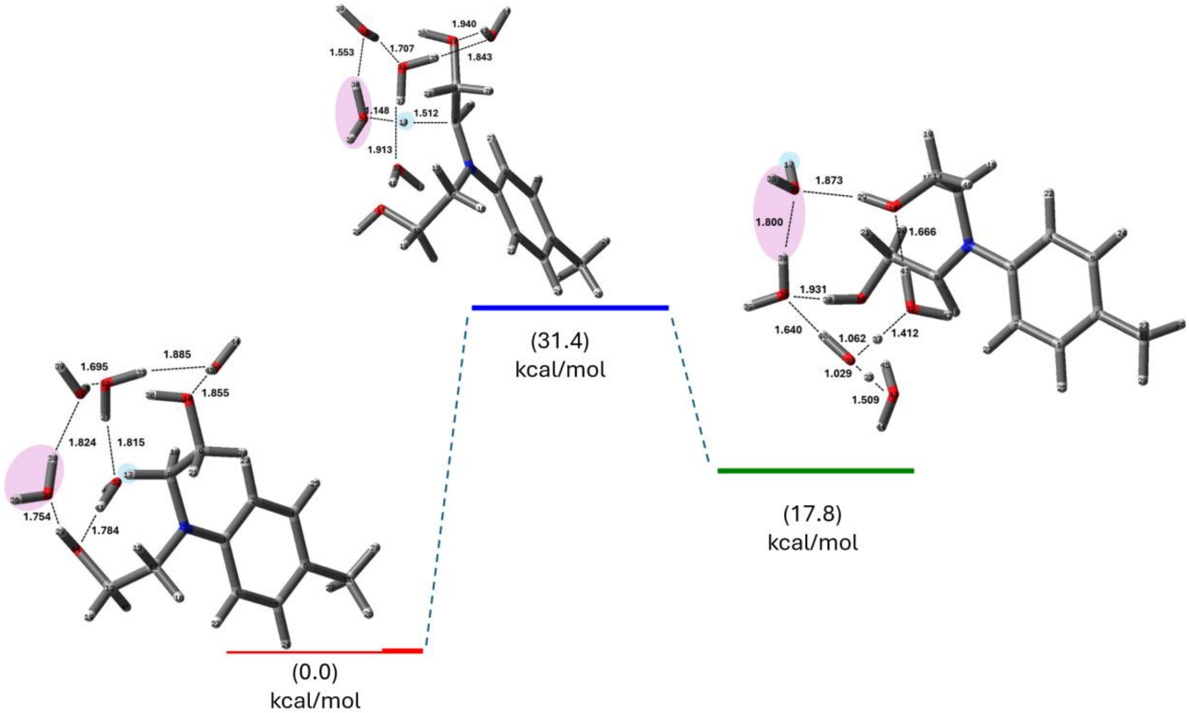

Figure S5. Reactant, transition state, and product structures for the PIDE$^{•+}$ deprotonation pathway along with the corresponding Gibbs free energy differences (kcal·mol^{-1}) relative to the reactant for both the transition state and the product. From M062X-D3-PCM/def2-TZVP calculations including five explicit water molecules. The main water molecule involved in the reaction is highlighted in pink, while the transferred proton is highlighted in light blue.

Electrochemiluminescence

ECL microscopy

The imaging was performed in the a-Clipse C electrochemical cell (Idylle Labs, Fig. S6), comprising either GC or Pt electrodes (A = 0.071 cm^2) where Ru(II)-functionalized beads were drop-cast, Pt counter, and Ag/AgCl (4 M KCl gel) reference electrodes using solutions of 0.3 M PB with 100 mM of either TPrA, BIDE or BIS-TRIS (pH 6.8) and a solution of 0.3 M acetate buffer with 100 mM PIDE at pH 5. The different solutions were inserted in the electrochemical cell with a pressure-driven flow controller (OB1 Mk3, Elveflow) equipped with a flux sensor (Flow-04D working range from 0 to 1000 μL/min) and exchanged, when necessary, with a 10-way bidirectional valve (MUX distributor). For microscopic imaging, a ECLIPSE Ti2 inverted microscope from Nikon (Chiyoda, Tokyo, Japan) equipped with an ultrasensitive EMCCD camera (EM-CCD 9100−13 from Hamamatsu, Japan) was used with a resolution of 512 × 512 pixel and a size of 16 × 16 μm^2. The microscope was enclosed in a homemade dark box to avoid interferences from external light. The images were acquired with air objective from Nikon (magnification 100×/NA 0.8/WD (mm) 4.5). Additionally, the integrated system included a SP-300 potentiostat (BioLogic Science Instrument, France) triggered with the camera.

ECL images were captured at 200 ms intervals during a double chronoamperometric pulse: open circuit potential for 2 s, followed by 1.7 V (*vs* Ag/AgCl 4M KCl) for 13 s.

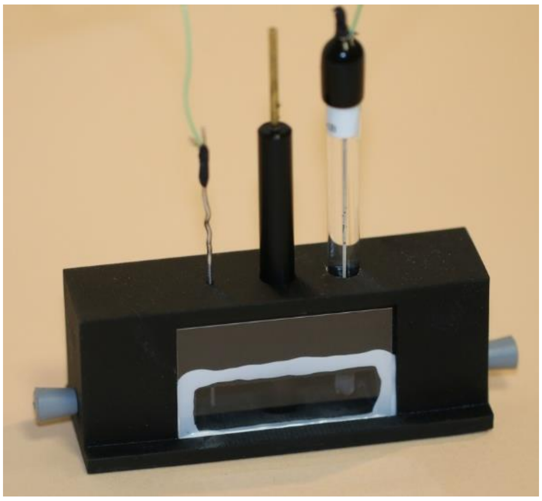

Figure S6. Photograph of the a-Clipse C electrochemical cell used for ECL measurements, showing the working, counter, and reference electrode configuration. Image courtesy of Idylle Labs.

Data elaboration: Integration of the ECL signal

The ECL intensity, whether from a given frame of a transient measurement or a single integrated image, was calculated over a circular ROI (r = 2.62 μm, A = 21.5 μm^2) centered on the bead (Fig. S7). The raw ECL value was obtained by summing the ECL intensity of all pixels within the ROI (i.e., integration). During data processing, the background noise, arising from the thermal dark current of the EM-CCD camera at its operating temperature (-45 °C), was subtracted from the raw averaged ECL. In particular, the background noise was determined by integrating the signal over a 21.5 μm^2 ROI centered on a region where no bead emission is detected.

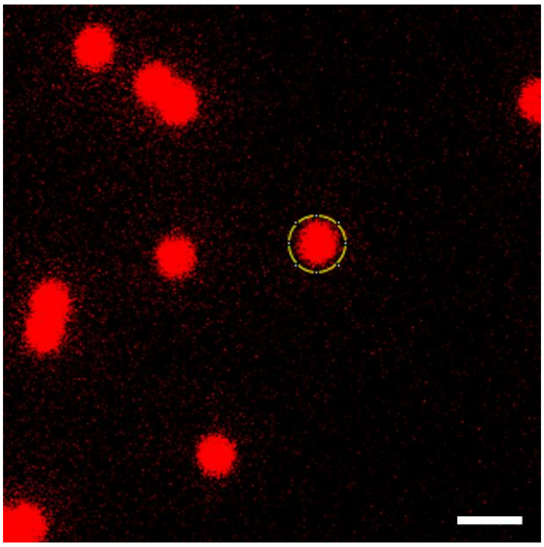

Figure S7. Model ECL image of data processing representing the 21.5 μm^2 ROI used for raw ECL and background quantification. Scale bar, 5 μm.

Depth of field

The depth of field (DOF) represents the thickness of a virtual layer within which objects appear acceptably sharp in an image. Generally, the total DOF is given by the sum of the wave and geometrical optical depths of fields as:

$$d_{\mathrm{tot}} = \frac{\lambda \cdot n}{\mathrm{NA}^2} + \frac{2nb}{\mathrm{M} \cdot \mathrm{NA}}$$

Where d_{tot} represents the total DOF, λ is the wavelength of emission (λ = 0.610 μm for $[Ru(bpy)_3]^{2+}$), n is the refractive index of the medium between the coverslip and the objective lens (n_{air} = 1), and NA equals the objective numerical aperture (NA = 0.8). The variable b is the basic pixel size of the EM-CCD camera (0.15871 μm) under 100× magnification (M).

Under these conditions, d_{tot} = 0.96 μm. Although this DOF accounts for approximately just a third of the thickness of the bead, it has been demonstrated that the ECL image integrates photons also from layers far away from the focal plane.[12]

ECL imaging on GC electrode

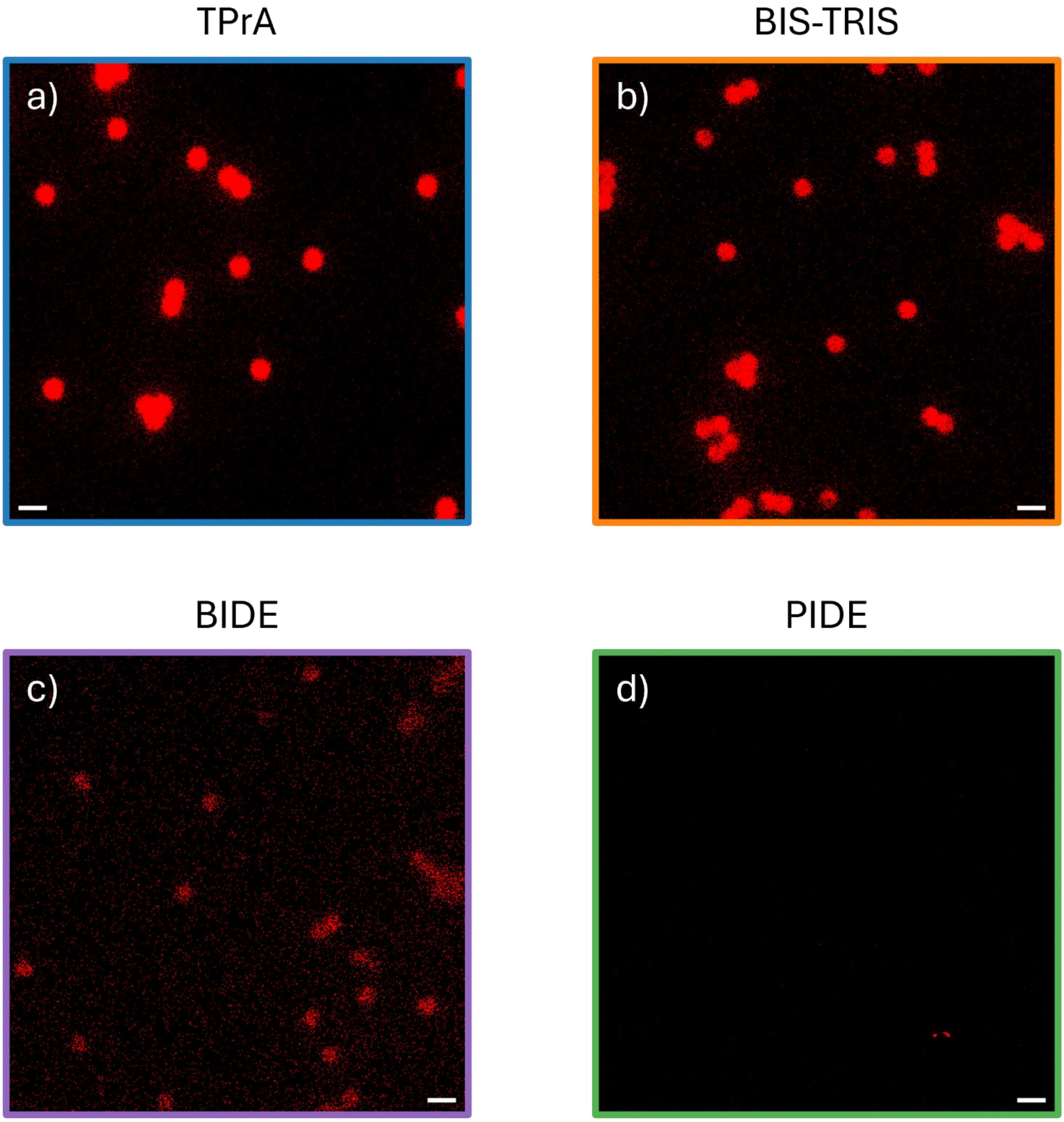

Figure S8. ECL images of $[Ru(bpy)_3]^{2+}$-functionalized beads in 0.3 M PB with 0.1 M (a) TPrA, (b) BIS-TRIS, or (c) BIDE (pH 6.8) or in (d) 0.3 M acetate buffer with 0.1 M PIDE (pH 5). The images were captured on a GC electrode with an EM-CCD camera during a two-step chronoamperometry measurement: the ECL signal was recorded for 4 s while holding the electrode at 1.7 V *vs* Ag/AgCl, after a 2 s step at open circuit potential. Magnification, ×100; objective numerical aperture, 0.8; gain, 5; sensitivity, 1200; scale bar, 5 μm. For each image, the contrast scale was adjusted to maximize the signal-to-noise ratio and are not meant then for comparative purpose. Comparative ECL images are reported in Fig. 3. A red lookup table was applied to the native greyscale images to generate false-color images resembling the emission of $[Ru(bpy)_3]^{2+}$.

COMSOL simulations

Finite element simulations were carried out on a single bead by the commercial software COMSOL Multiphysics (Version 6.1). In this simulation, a 2D axisymmetric geometry (h = 40 μm × r_L = 4.2 μm) was built to simulate the physical geometry of the substrate used in ECL experiments (Fig. S9a), with a refined mesh at the electrode surface and the bead surface boundaries (Fig. S9b). The bulk boundary is set at a constant concentration of coreactant (100 mM). "Transport of Diluted Species" physical field was employed for studying the time-dependent transport of diluted species involved in ECL reactions. Instead, $[Ru(bpy)_3]^{2+}$ labels attached to the bead surface and reactions at the bead/electrolyte interface are described in the "Surface Reactions" physical field.

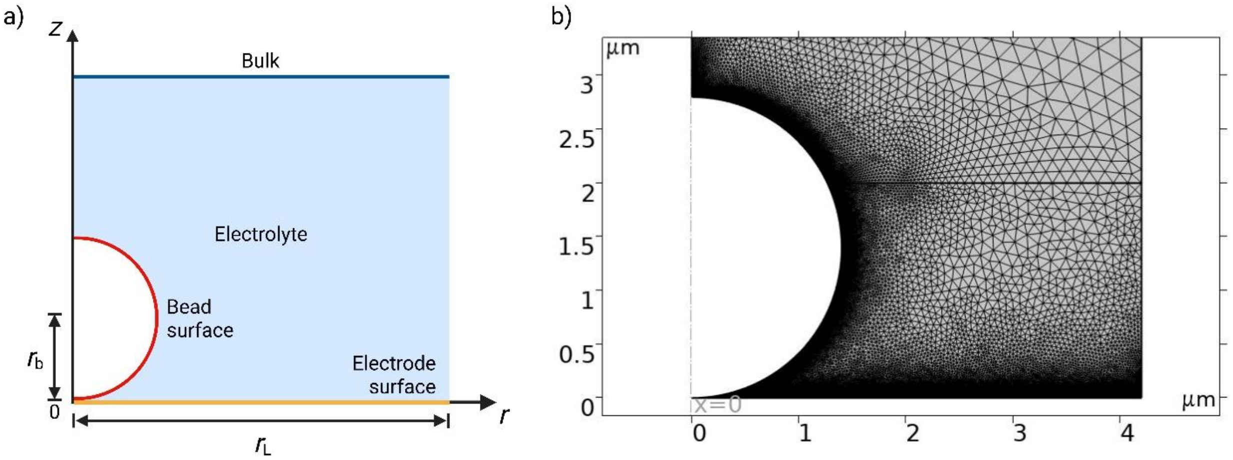

Figure S9. a) 2D axisymmetric model employed in the COMSOL simulation (not to real scale). r and z coordinates represent the parallel and normal directions to the electrode surface, respectively. r_b represents the radius of the bead. r_L represents the length of the electrode. b) Mesh settings for COMSOL simulation with refinements at the electrode and bead boundaries.

The reactive species include the redox-active states of the luminophore and the coreactant involved in the generation of the ECL signal. Namely, the simulation encompasses $[Ru(bpy)_3]^{2+}$, its reduced form, $[Ru(bpy)_3]^{+}$, and the excited $[Ru(bpy)_3]^{2+*}$ on the surface of the bead; while the neutral coreactant and its protonated form, its radical cation, and the neutral radical are freely diffusing in solution.

The acid–base pre-equilibrium between the tertiary amine (B) and the buffer acid (PBH) was modeled according to the general reaction scheme:

$$\mathrm{B} + PBH \rightleftharpoons \mathrm{BH}^+ + \mathrm{PB}$$

Proton transfer between the amine and buffer species was assumed to be rapid and diffusion-controlled in aqueous solution, with an intrinsic proton-transfer rate constant k_{DL} on the order of 10^{10} $M^{-1}\cdot s^{-1}$, consistent with previous modeling by Eigen.[13,14] The microscopic forward proton-transfer rate was expressed as:

$$r_f = k_{\mathrm{DL}}[B][PBH]$$

The concentrations of reactive species were related to total concentrations as:

$$[B] = f_B[B]_{\mathrm{tot}}, [PBH] = f_{HA}[PB]_{\mathrm{tot}}$$

Where f_B and f_{PBH} represent the fractions of unprotonated amine and protonated buffer, respectively. Their fractional abundances were calculated using the Henderson–Hasselbalch relationship:

$$f_B = \frac{1}{1 + 10^{\mathrm{p}K_a(BH^+) - \mathrm{pH}}}$$
$$f_{HA} = \frac{1}{1 + 10^{\mathrm{pH} - \mathrm{p}K_a(HA)}}$$

Substitution of the speciation relationships yields the observed forward rate:

$$k_f = k_{\mathrm{DL}} f_B f_{HA}$$

This formulation accounts for pH-dependent acid–base speciation within the kinetic model. The backward rate constant was obtained from the equilibrium constant for proton transfer:

$$K_{\mathrm{eq}} = 10^{\mathrm{p}K_a(BH^+) - \mathrm{p}K_a(HA)}$$

and detailed balance:

$$k_b = \frac{k_f}{K_{\mathrm{eq}}}$$

This ensures thermodynamic consistency between forward and backward reactions.

Following oxidation of tertiary amines, the simulation encompasses their radical cations and neutral radicals. Equations describing all the electrochemical and chemical reaction steps incorporated in the model are provided in Table S3. All parameters used in the simulation are summarized in Table S4.

Table S3. Reactions involved in the simulation

Category	Reaction Equation	
Charge Transfer Reactions	$\mathrm{TPrA} \xrightarrow{k_0} \mathrm{TPrA}^{\bullet+} + e^-$	(1)
	$\mathrm{TPrA}^{\bullet} \xrightarrow{k_0} \mathrm{Im}^+ + e^-$	(2)
Homogeneous Reactions	$\mathrm{TPrA} + \mathrm{PBH}^+ \underset{k_1}{\overset{k_{1-}}{\rightleftarrows}} \mathrm{TPrAH}^+ + \mathrm{PB}$	(3)
	$\mathrm{TPrA}^{\bullet+} \underset{k_3}{\overset{k_{3-}}{\rightleftarrows}} \mathrm{TPrA}^{\bullet} + H^+$	(4)
	$\mathrm{PBH}^+ \underset{k_{prot}}{\overset{k_{dep}}{\rightleftarrows}} \mathrm{PB} + H^+$	(5)
	$\mathrm{TPrA}^{\bullet+} + \mathrm{TPrA}^{\bullet} \xrightarrow{k_d} \mathrm{TPrA} + \mathrm{Im}^+$	(6)
Reaction on the bead surface	$[\mathrm{Ru(bpy)_3}]^{2+} + \mathrm{TPrA}^{\bullet} \xrightarrow{k_5} [\mathrm{Ru(bpy)_3}]^{+} + \mathrm{Im}^+$	(7)
	$[\mathrm{Ru(bpy)_3}]^{+} + \mathrm{TPrA}^{+\bullet} \xrightarrow{k_5} [\mathrm{Ru(bpy)_3}]^{2+*} + \mathrm{TPrA}$	(8)
	$[\mathrm{Ru(bpy)_3}]^{2+*} \xrightarrow{k_{em}} [\mathrm{Ru(bpy)_3}]^{2+} + h\nu$	(9)

Table S4. A summary of simulation parameters

Name	Value	Description
alpha	0.5	Electron transfer coefficient

F	96485 [$C\cdot mol^{-1}$]	Faraday constant
R	8.314 [$J\cdot mol^{-1}\cdot K^{-1}$]	Gas constant
T	298.15 [K]	Temperature
f_a	38.92 [V^{-1}]	F/RT
n	1	n° of electrons exchanged
N_a	$6.02\cdot 10^{23}$ [mol^{-1}]	Avogadro number
E_a	0.95 [V]	Applied potential
E^o	0.9 [V]	TPrA standard oxidation potential
E^o_d	-1.7 [V]	TPrA radical standard oxidation potential
D	$5\cdot 10^{-6}$[$cm^2\cdot s^{-1}$]	Diffusion coefficient for TPrA and its radicals
D_H	$5\cdot 10^{-5}$[$cm^2\cdot s^{-1}$]	Diffusion coefficient for H^+
D_{min}	$1\cdot 10^{-50}$ [$cm^2\cdot s^{-1}$]	Diffusion coefficient for hν
k_o	Variable	Rate constant at zero potential for coreactant ET
$k_{1\_}$	(k1_/K_{eq})	Forward rate constant Eq. 3
k_1	(k_{DL}*(1/(1+K_{base}))*(1/(1+K_{acid})))	Backward rate constant Eq. 3
$k_{3\_}$	Variable	Forward rate constant Eq. 4
k_3	Variable	Backward rate constant Eq. 4
k_5	$1\cdot 10^6$ [$M^{-1}\cdot s^{-1}$]	Rate constant Eq. 7
k_d	$1\cdot 10^9$ [$M^{-1}\cdot s^{-1}$]	Rate constant Eq. 6
k_{em}	$1\cdot 10^8$ [s^{-1}]	Photon emission rate
pK_a	Variable	pK_a amine
pK_{PB}	7.2	pK_a phosphate buffer
pH	6.8	
H_0	$1.58\cdot 10^{-7}$ [M]	Initial [H^+] at pH 6.8
A_{0t}	0.1[M]	Initial [coreactant]
A_0	$10^{pH\text{-}pKa}\times 100 / 10^{pH\text{-}pKa}+1$	Concentration of unprotonated coreactant
CC_0	A_{0t}-A_0	Concentration of protonated coreactant
CPBH	0.215 [M]	Concentration of protonated phosphate
CPB	0.085 [M]	Concentration of unprotonated phosphate
k_{prot}	$1\cdot 10^{10}$ [$M^{-1}\cdot s^{-1}$]	Forward rate constant Eq. 5
k_{dep}	620 [s^{-1}]	Backward rate constant Eq. 5
C_0	0	
Ru_0	$9.67\cdot 10^{-6}$ [$mol\cdot m^{-2}$]	Initial concentration of [$Ru(bpy)_3$]$^{2+}$ on the bead
R_b	1.4[μm]	Bead radius
k_{DL}	1e10 [$M^{-1}\cdot s^{-1}$]	Diffusion-limited rate constant
f_{base}	pK_a-pH	
K_{base}	10^{fbase}	
f_{acid}	pH-pK_{PB}	
K_{acid}	10^{facid}	
K_{eq}	$10^{pKa\text{-}pKPB}$	

The rate constants for heterogeneous oxidation at the studied overpotential (E_a), modeled according to Butler-Volmer laws, are presented in Table S5.

Table S5. Butler-Volmer equations for determining the rate constants of electrode reactions of electroactive species.

Name	Butler-Volmer Equation	Description
kI	$k_o \cdot exp((1\text{-}alpha) \cdot f_a \cdot (E_a\text{-}E^o))$	Forward constant for coreactant oxidation
kA	$k_o \cdot exp(\text{-}alpha \cdot f_a \cdot (E_a\text{-}E^o))$	Backward constant for coreactant oxidation
kX	$k_o \cdot exp((1\text{-}alpha) \cdot f_a \cdot (E_a\text{-}E^o{}_d))$	Forward constant for radical oxidation

The inward flux (J) at the electrode surface can be expressed as follows in Eqs. 10-11:

$$J_{TPrA} = -J_{TPrA^{\bullet+}} = kI \cdot [TPrA^{\bullet+}] - kA \cdot [TPrA] \quad (10)$$

$$J_{TPrA^{\bullet}} = -\, kE \cdot [TPrA^{\bullet}] \quad (11)$$

where [TPrA], [$TPrA^{\cdot+}$], and [$TPrA^{\cdot}$] represent the concentrations of coreactant, radical cation, and neutral radical, respectively.

On the other hand, J at the bead surface upon consumption of coreactant radicals are presented in Eqs. 12-14:

$$J_{Ru^{+}} = k_5 \cdot [TPrA^{\bullet}] \cdot \left[Ru^{2+}{}_{(bound)}\right] - k_5 \cdot [TPrA^{\bullet+}] \cdot \left[Ru^{+}{}_{(bound)}\right] \quad (12)$$

$$J_{Ru^{2+*}} = k_5 \cdot [TPrA^{\bullet+}] \cdot \left[Ru^{+}{}_{(bound)}\right] - k_{em} \cdot \left[Ru^{2+*}{}_{(bound)}\right] \quad (13)$$

$$J_{Ru^{2+}} = k_{em} \cdot \left[Ru^{2+*}{}_{(bound)}\right] - k_5 \cdot [TPrA^{\bullet}] \cdot \left[Ru^{2+}{}_{(bound)}\right] \quad (14)$$

where [$Ru^{2+}{}_{(bound)}$], [$Ru^{+}{}_{(bound)}$], and [$Ru^{2+*}{}_{(bound)}$] represent the concentrations of $[Ru(bpy)_3]^{2+}$, $[Ru(bpy)_3]^{+}$, and $[Ru(bpy)_3]^{2+*}$ on the bead surface; while [$TPrA^{\cdot}$] and [$TPrA^{\cdot+}$] represent the concentrations of coreactant neutral radical and radical cation, respectively.

The upper boundary in the model (Bulk in Fig. S9a) represent the bulk solution where the initial concentrations remain constant throughout the simulation, promoting diffusion along the z-axis. Isotropic diffusion of species in solution is formulated according to the Fick's second law (Eq. 15), assuming that the contribution from the migration and convection is negligible:

$$\frac{\partial c_i}{\partial t} = D_i \Delta^2 c_i + R_i \quad (15)$$

where c_i and D_i represent the local concentration and diffusion coefficient of ith species, respectively, while t is the time, Δ represents the Laplacian, and R_i is the reaction flux necessary to maintain equilibrium.

We approximated the diffusion coefficient for the coreactant and its radicals to be $5 \cdot 10^{-10}$ $m^2 \cdot s^{-1}$ (D). A faster diffusion coefficient of $5 \cdot 10^{-9}$ $m^2 \cdot s^{-1}$ was assigned to the proton H^+ (D_H) and, eventually, the slowest coefficient of $5 \cdot 10^{-54}$ $m^2 \cdot s^{-1}$ was attributed to hν (D_{min}).

The concentration change of every species involved in the ECL process as a consequence of Eqs. 16-26 are described in Table S6.

Table S6. Concentration changes of every species involved in the ECL process.

Concentration change	Expression	
$\frac{\partial[TPrAH^+]}{\partial t}$	$k_{1\_}\cdot[TPrA]\cdot[PBH^+]$ - $k_1\cdot[PB]\cdot[TPrAH^+]$	16
$\frac{\partial[TPrA]}{\partial t}$	$k_1\cdot[TPrAH^+]_\_\cdot[PB]$ - $k_{1\_}\cdot[TPrA]\cdot[PBH^+]$ + $k_d\cdot[TPrA^{\bullet+}]\cdot[TPrA^\bullet]$ + $k_5\cdot[Ru^+]\cdot[TPrA^{\bullet+}]$	17
$\frac{\partial[TPrA^{\bullet+}]}{\partial t}$	$k_3\cdot[TPrA^\bullet]\cdot[H^+]$ - $k_{3\_}\cdot[TPrA^{\bullet+}]$ - $k_5\cdot[TPrA^{\bullet+}]\cdot[TPrA^\bullet]$ - $k_5\cdot[Ru^+]\cdot[TPrA^{\bullet+}]$	18
$\frac{\partial[TPrA^\bullet]}{\partial t}$	$k_{3\_}\cdot[TPrA^{\bullet+}]$ - $k_3\cdot[TPrA^\bullet]\cdot[H^+]$ - $k_5\cdot[TPrA^{\bullet+}]\cdot[TPrA^\bullet]$ - $k_5\cdot[Ru^{2+}]\cdot[TPrA^\bullet]$	19
$\frac{\partial[H^+]}{\partial t}$	$k_{3\_}\cdot[TPrA^{\bullet+}]$ - $k_3\cdot[TPrA^\bullet]\cdot[H^+]$ + $k_{dep}\cdot[PBH^+]$ - $k_{prot}\cdot[PB]\cdot[H^+]$	20
$\frac{\partial[PBH^+]}{\partial t}$	$k_1\cdot[PB]\cdot[TPrAH^+]$ - $k_{1\_}\cdot[TPrA]\cdot[PBH^+]$ + $k_{prot}\cdot[PB]\cdot[H^+]$ - $k_{dep}\cdot[PBH^+]$	21
$\frac{\partial[PB]}{\partial t}$	$k_{1\_}\cdot[TPrA]\cdot[PBH^+]$ - $k_1\cdot[PB]\cdot[TPrAH^+]$ + $k_{dep}\cdot[PBH^+]$ - $k_{prot}\cdot[PB]\cdot[H^+]$	22
$\frac{\partial[Ru^{2+}]}{\partial t}$	$k_{em}\cdot[Ru^{2+}*]$ - $k_5\cdot[Ru^{2+}]\cdot[TPrA^\bullet]$	23
$\frac{\partial[Ru^+]}{\partial t}$	$k_5\cdot[Ru^{2+}]\cdot[TPrA^\bullet]$ - $k_{IG1}\cdot[Ru^+]\cdot[TPrA^{\bullet+}]$	24
$\frac{\partial[Ru^{2+*}]}{\partial t}$	$k_5\cdot[Ru^+]\cdot[TPrA^{\bullet+}]$ - $k_{em}\cdot[Ru^{2+}*]$	25
$\frac{\partial[h\nu]}{\partial t}$	$k_{em}\cdot[Ru^{2+}*]$	26

Simulated $[Ru(bpy)_3]^{2+*}$ profiles

Although inclusion of reversible proton transfer in the simulations leads to a modest broadening of the excitation profile, the resulting thickening of the emitting layer remains limited and cannot be resolved under typical experimental ECL imaging or electrochemical conditions. Consequently, the system effectively operates in a deprotonation-dominated regime, consistent with the behavior observed experimentally. Within the kinetic framework (Eqs. 1-5 in the main text), backward protonation is expected to become significant only under more strongly acidic conditions, where proton availability is substantially increased. Moreover, in the present simulations, the neutral α-amino radical is treated as chemically stable and is not subjected to secondary decomposition pathways. This approximation likely overestimates the extent of radical recycling and, in turn, the impact of reversible proton transfer on the spatial emission profile. Taken together, these considerations support the conclusion that, under practical ECL conditions, proton transfer can be reasonably treated as effectively irreversible.

PIDE characterization

Radical cations of aniline derivatives, such as N,N-dimethylaniline, have been shown to delocalize the unpaired electron density over the aromatic group via mesomeric effect, particularly at the ortho and para positions.[15,16] This resonance substantially stabilizes the electrogenerated radical cation compared to alkyl analogues. Yet, N,N-dimethylaniline is poorly soluble in water and its delocalized spin density promotes dimerization between radical cations, similar to diphenylamine derivatives.[17–19] To address these issues, a new tertiary amine—PIDE (Fig. S10a-c)—is introduced as a model coreactant: the ethanol moieties introduce affinity to aqueous phase and possible hydrogen-bond stabilization in water; while the para methyl group suppresses dimerization. The ortho position, instead, is assumed to be unreactive by sterical hindrance. Since the structural modifications compared to N,N-dimethylaniline exert a minor impact on the electronic properties, the key radical-stabilizing properties are expected to remain similar (Fig. S10d).

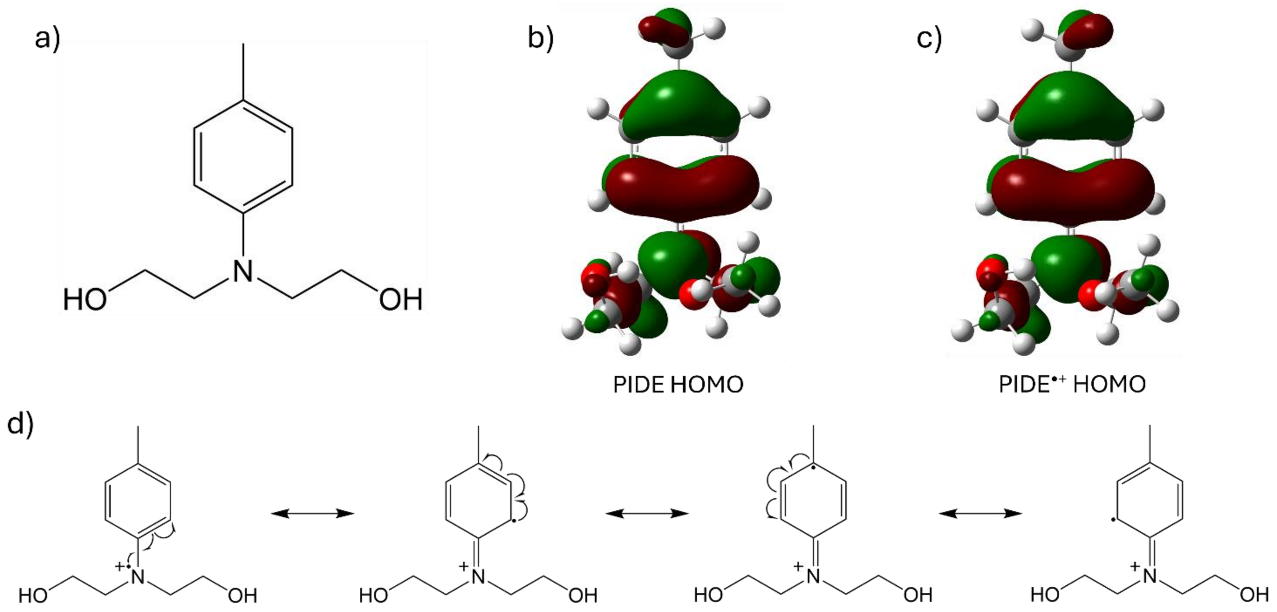

Figure S10. a) Chemical structure of PIDE. b) Contour plot of HOMO for PIDE. c) Contour plot of HOMO for PIDE$^{•+}$. Molecular geometries of b) and c) were optimized via DFT calculations at the M062X-D3-PCM/def2SVP level of theory in implicit water. d) Resonance structure of PIDE$^{•+}$ involved in the delocalization of the unpaired electron.

The NBO and Multiwfn analyses indicate that oxidation of the coreactant primarily involves the nitrogen lone pair, but that the resulting radical cation is efficiently stabilized by redistribution of the resulting hole over the conjugated framework, which in turn hinders α-deprotonation. In the neutral species, the HOMO is largely dominated by the nitrogen lone pair, with a strong contribution from N-centered p-type orbitals (~33%), consistent with a readily oxidizable electron-donating site. Although the pK_a of PIDE has not been reported in the literature, a closely related analogue, N-phenyl diethanolamine, exhibits a pK_a of approximately 4.3, indicating markedly reduced basicity. This low basicity is consistent with substantial delocalization of the nitrogen lone pair into the aromatic π system. Upon one-electron oxidation, this nitrogen contribution decreases to ~20%, and the corresponding singly occupied molecular orbital becomes preferentially distributed over the aromatic carbon ring. At the same time, the orbital delocalization index decreases (17.29 → 13.28), indicating a reorganization toward a more selective and uneven localization within

the π system rather than a uniform delocalization over the entire molecule. This redistribution reduces the degree of positive charge and spin density localized at the N–Cα region, limiting polarization of the adjacent Cα–H bonds and thereby suppressing their acidity. Consequently, although the initial oxidation is triggered by removal of electron density from the nitrogen lone pair, the radical cation is subsequently stabilized by π delocalization over the aromatic backbone, which lowers its reactivity and disfavors α-deprotonation pathways. This electronic structure promotes the formation of a relatively persistent radical cation.
These computational observations are supported by experimental electrochemical evidence. PIDE was investigated by cyclic voltammetry (CV) at varying scan rates to assess the stability and reversibility of the oxidized intermediate (Fig. S11).

PIDE measurements were performed at pH 5 due to solubility limitations in neutral aqueous media. At this pH, partial protonation ensures sufficient dissolution while maintaining a significant fraction of the amine in its unprotonated, electroactive form. This condition represents the highest pH at which reliable and reproducible measurements could be obtained.

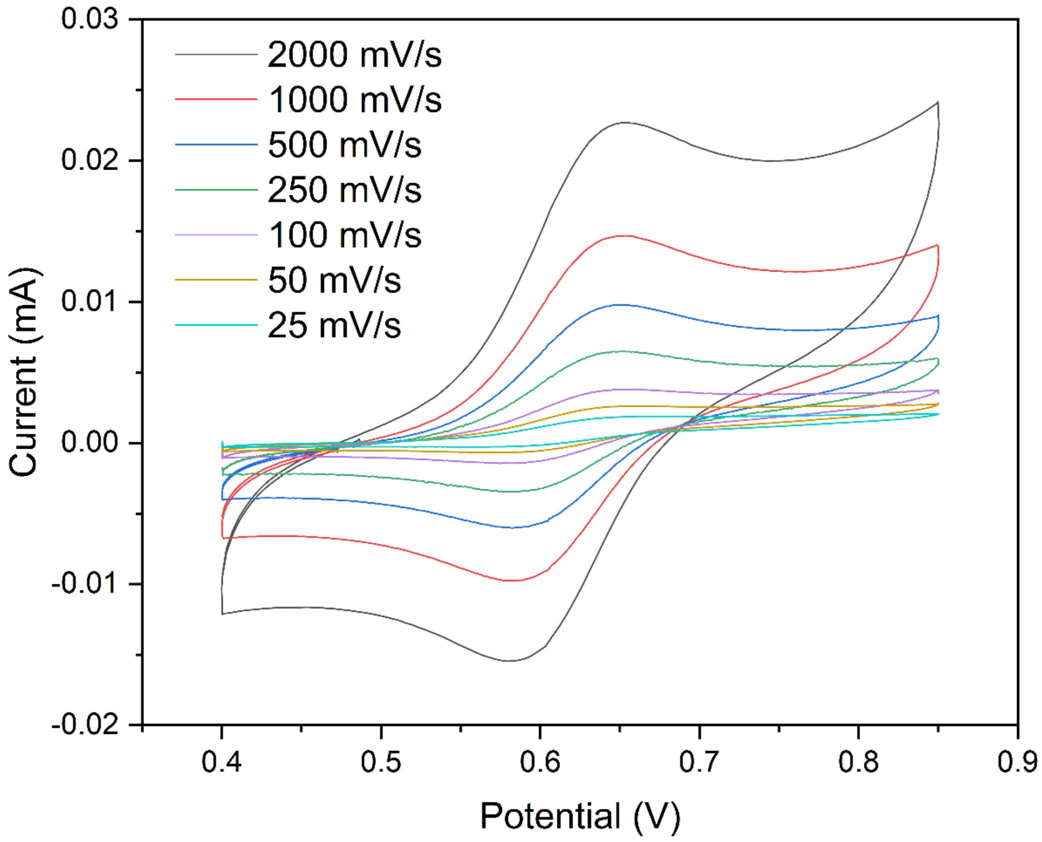

Figure S11. Cyclic voltammograms of 1 mM PIDE in acetate buffer 0.3 M (pH 5) at different scan rates, from 2000 mV/s (grey line) to 25 mV/s (cyan line). The measurements were performed using a three-electrode setup including a GC working electrode (d = 1 mm), a Ag/AgCl reference electrode and a Pt wire counter electrode.

At potentials above approximately 0.5 V *vs* Ag/AgCl, PIDE undergoes a one-electron oxidation that removes an electron from the nitrogen lone pair, producing the N-centered radical cation $PIDE^{\bullet+}$. Notably, the oxidation potential is anticipated compared to alkyl amines: in this case, the non-bonding electrons are less tightly held as predicted by delocalization of the non-bonding orbital (Fig. S10b).

The oxidation process is nearly fully reversible at a scan rate of 2000 mV/s, but progressively approaches irreversibility as the scan rate is slowed down until no reduction current associated to PIDE$^{\bullet+}$ is observed at 50 mV/s. This pronounced scan rate dependence indicates the formation of a long-lived radical cation that undergoes a slow follow-up deprotonation to PIDE$^{\bullet}$ on the experimental timescale.

Tafel analysis and determination of electron-transfer kinetics

Steady-state polarization curves were recorded under charge-transfer-controlled conditions. The measured currents were converted to current densities by normalization to the geometric electrode area. The overpotential (η) was calculated as the difference between the applied potential and the onset potential (E_0, i.e., the intersection point between the tangent to faradaic zone and the x-axis), since the standard potential could not be determined.

Tafel plots were constructed by plotting η as a function of the decimal logarithm of the absolute current density, $\log_{10}|j|$. Linear regions corresponding to kinetically controlled anodic processes were identified and fitted by linear regression. The exchange current density (j_0) was determined by extrapolating the linear Tafel region to zero overpotential (η = 0). Note that, in the Tafel analysis, the η range was limited to working potentials below 1.4 V to exclude the contribution of electrochemical oxidation of water. The standard heterogeneous electron-transfer rate constant (k^0) was then calculated from:

$$j_0 = nFk^0C_O^{1-\alpha}C_R^{\alpha}$$

where C_O and C_R are the bulk concentrations of oxidized and reduced species, respectively. For systems with equal bulk concentrations, this expression simplifies to:

$$k^0 = \frac{j_0}{nFC^*}$$

where C^* is the effective concentration of tertiary amine calculated according to the Henderson–Hasselbalch equation. All measurements were performed at $T = 298$ K.

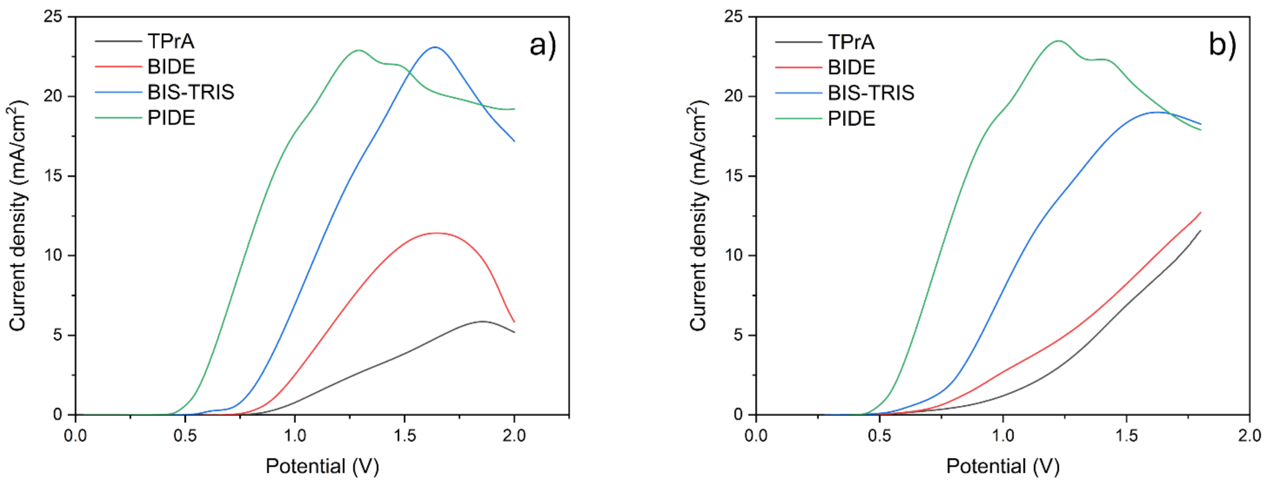

Figure S12. Linear sweep voltammograms of 0.1 M TPrA (grey curves), BIDE (red curves), and BIS-TRIS (blue curves) recorded in 0.3 M PB (pH 6.8), and 0.1 M PIDE (green curves) recorded in 0.3 M acetate buffer (pH 5), measured at (a) GC and (b) Pt electrodes. The scan rate was 100 mV·s^{-1}.

To estimate the concentration of radical cations generated at the electrode surface, finite element simulations were performed and the interfacial concentration was evaluated 0.5 s after the potential sweep. An overpotential of 0.05 V was applied in the simulations in order to ensure that the oxidation process remained within the charge-transfer-controlled regime.

At higher overpotentials, the oxidation current becomes progressively limited by mass transport and follow-up chemical reactions, leading to diffusion-controlled concentration profiles that are largely insensitive to the intrinsic heterogeneous electron-transfer rate constant. Such conditions are therefore unsuitable for comparing interfacial kinetics between different electrode materials.

By restricting the overpotential to 0.05 V, the simulated current remains governed by Butler–Volmer kinetics, and the resulting radical cation concentration directly reflects the heterogeneous oxidation rate at the electrode surface. This approach allows meaningful comparison of the intrinsic oxidation efficiencies of the investigated amines on different electrode materials.

Table S7. Standard potential (E^0), rate constant of oxidation at zero potential (k^0), and radical cation concentration at the electrode surface after 0.5 s ($[NR_3^{\bullet+}]$) for all the investigated amines on either GC or Pt electrode.

Electrode material		TPrA	BIDE	BIS-TRIS	PIDE
	E^0 [V]	0.87	0.86	0.77	0.52
GC	k^0 [cm/s]	$4.98\cdot10^{-1}$	$4.97\cdot10^{-2}$	$9.63\cdot10^{-4}$	$1.19\cdot10^{-3}$
	$[NR_3^{\bullet+}]$ [M]	$6.24\cdot10^{-5}$	$1.23\cdot10^{-4}$	$2.60\cdot10^{-3}$	$4.9\cdot10^{-2}$
	E^0 [V]	0.75	0.7	0.73	0.53
Pt	k^0 [cm/s]	$1.59\cdot10^{-1}$	$1.96\cdot10^{-2}$	$9.91\cdot10^{-4}$	$1.38\cdot10^{-3}$
	$[NR_3^{\bullet+}]$ [M]	$3.93\cdot10^{-5}$	$7.10\cdot10^{-5}$	$2.65\cdot10^{-3}$	$5.2\cdot10^{-2}$

ECL imaging on Pt electrode

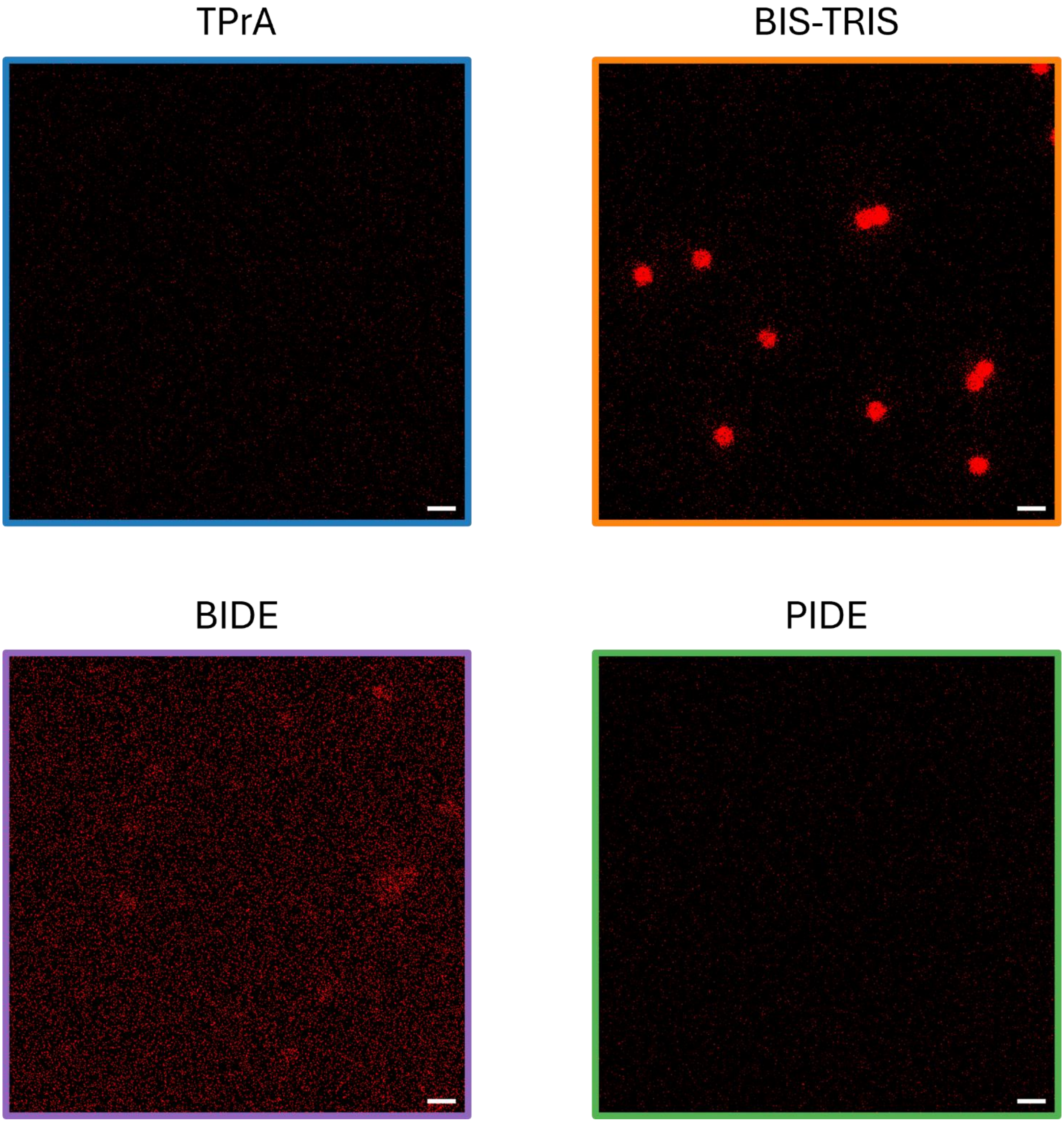

Figure S13. ECL images of $[Ru(bpy)_3]^{2+}$-functionalized beads in 0.3 M PB with 0.1 M (a) TPrA, (b) BIS-TRIS, or (c) BIDE (pH 6.8) or in (d) 0.3 M acetate buffer with 0.1 M PIDE (pH 5). The images were captured on a Pt electrode with an EM-CCD camera during a two-step chronoamperometry measurement: the ECL signal was recorded for 1 s while holding the electrode at 1.7 V vs Ag/AgCl, after a 2 s step at open circuit potential. Magnification, ×100; objective numerical aperture, 0.8; gain, 5; sensitivity, 1200; scale bar, 5 μm. For each image, the contrast scale was adjusted to maximize the signal-to-noise ratio and are not meant then for comparative purpose. Comparative ECL images are reported in Fig. 3. A red lookup table was applied to the native greyscale images to generate false-color images resembling the emission of $[Ru(bpy)_3]^{2+}$.

References

(1) Frisch, M. J.; Trucks, G. W.; Schlegel, H. B.; Scuseria, G. E.; Robb, M. A.; Cheeseman, J. R.; Scalmani, G.; Barone, V.; Petersson, G. A.; Nakatsuji, H.; Li, X.; Caricato, M.; Marenich, A.; Bloino, J.; Janesko, B. G.; Gomperts, R.; Mennucci, B.; Hratchian, H. P.; Ortiz, J. V.; Izmaylov, A. F.; Sonnenberg, J. L.; Williams-Young, D.; Ding, F.; Lipparini, F.; Egidi, F.; Goings, J.; Peng, B.; Petrone, A.; Henderson, T.; Ranasinghe, D.; Zakrzewski, V. G.; Gao, J.; Rega, N.; Zheng, G.; Liang, W.; Hada, M.; Ehara, M.; Toyota, K.; Fukuda, R.; Hasegawa, J.; Ishida, M.; Nakajima, T.; Honda, Y.; Kitao, O.; Nakai, H.; Vreven, T.; Throssell, K.; Montgomery, J. A.; Peralta, J. E.; Ogliaro, F.; Bearpark, M.; Heyd, J. J.; Brothers, E.; Kudin, K. N.; Staroverov, V. N.; Keith, T.; Kobayashi, R.; Normand, J.; Raghavachari, K.; Rendell, A.; Burant, J. C.; Iyengar, S. S.; Tomasi, J.; Cossi, M.; Millam, J. M.; Klene, M.; Adamo, C.; Cammi, R.; Ochterski, J. W.; Martin, R. L.; Morokuma, K.; Farkas, O.; Foresman, J. B.; Fox, D. J. Gaussian 09, Revision B.01. (Gaussian. Inc., Wallingford, 2016).

(2) Neese, F. Software Update: The ORCA Program System—Version 6.0. *Wiley Interdiscip. Rev. Comput. Mol. Sci.* **2025**, *15* (2), e70019.

(3) Bannwarth, C.; Ehlert, S.; Grimme, S. GFN2-XTB - An Accurate and Broadly Parametrized Self-Consistent Tight-Binding Quantum Chemical Method with Multipole Electrostatics and Density-Dependent Dispersion Contributions. *J. Chem. Theory Comput.* **2019**, *15* (3), 1652–1671.

(4) Ehlert, S.; Stahn, M.; Spicher, S.; Grimme, S. Robust and Efficient Implicit Solvation Model for Fast Semiempirical Methods. *J. Chem. Theory Comput.* **2021**, *17* (7), 4250–4261.

(5) Zhao, Y.; Truhlar, D. G. The M06 Suite of Density Functionals for Main Group Thermochemistry, Thermochemical Kinetics, Noncovalent Interactions, Excited States, and Transition Elements: Two New Functionals and Systematic Testing of Four M06 Functionals and 12 Other Functionals (T. *Theor. Chem. Acc.* **2008**, *120*, 215–241.

(6) Grimme, S.; Antony, J.; Ehrlich, S.; Krieg, H. A Consistent and Accurate Ab Initio Parametrization of Density Functional Dispersion Correction (DFT-D) for the 94 Elements H-Pu. *J. Chem. Phys.* **2010**, *132* (15), 20.

(7) Grimme, S.; Ehrlich, S.; Goerigk, L. Effect of the Damping Function in Dispersion Corrected Density Functional Theory. *J. Comput. Chem.* **2011**, *32* (7), 1456–1465.

(8) Tomasi, J.; Mennucci, B.; Cammi, R. Quantum Mechanical Continuum Solvation Models. *Chem. Rev.* **2005**, *105* (8), 2999–3093.

(9) Mennucci, B. Polarizable Continuum Model. *Wiley Interdiscip. Rev. Comput. Mol. Sci.* **2012**, *2* (3), 386–404.

(10) Zhang, X.; Jie, J.; Song, D.; Su, H. Deprotonation of Guanine Radical Cation G•+Mediated by the Protonated Water Cluster. *J. Phys. Chem. A* **2020**, *124* (29), 6076–6083.

(11) Pliego, J. R.; Riveros, J. M. The Cluster-Continuum Model for the Calculation of the Solvation Free Energy of Ionic Species. *J. Phys. Chem. A* **2001**, *105* (30), 7241–7247.

(12) Feng, Y.; Zhou, W.; Wang, X.; Zhang, J.; Zou, M.; Zhang, C.; Qi, H. Imaging and Simulation of Ruthenium Derivative Coating Microbeads at the Opaque Electrode with Electrogenerated Chemiluminescence. *Chem. Biomed. Imaging* **2023**, *1* (7), 648–658.

(13) Eigen, M.; De Maeyer, L. Self-Dissociation and Protonic Charge Transport in Water And. *Proc. R. Soc. London. Ser. A. Math. Phys. Sci.* **1958**, *247* (1251), 505–533.

(14) Eigen, M. Proton Transfer, Acid-Base Catalysis, and Enzymatic Hydrolysis. Part I: ELEMENTARY PROCESSES. *Angew. Chemie Int. Ed.* **1964**, *3* (1), 1–19.

(15) Raczynska, E. D.; Stepniewski, T. M.; Kolczynska, K. Consequence of One-Electron Oxidation and One-Electron Reduction for Aniline. *J. Mol. Model.* **2011**, *17* (12), 3229–3239.

(16) Zhang, S.; Wang, W.; Liu, S.; Sui, Y.; Zhang, Z. C.; Tan, G.; Sun, Q.; Wang, X. Putting Aniline Radical Cations in a Bottle. *Sci. China Chem.* **2017**, *60* (11), 1439–1443.
(17) Nelson, R. F.; Adams, R. N. Anodic Oxidation Pathways of Substituted Triphenylamines. II. Quantitative Studies of Benzidine Formation. *J. Am. Chem. Soc.* **1968**, *90* (15), 3925–3930.
(18) Blanchard, P.; Malacrida, C.; Cabanetos, C.; Roncali, J.; Ludwigs, S. Triphenylamine and Some of Its Derivatives as Versatile Building Blocks for Organic Electronic Applications. *Polym. Int.* **2019**, *68* (4), 589–606.
(19) Fracassa, A.; Calogero, F.; Pavan, G.; Nikolaou, P.; Fermi, A.; Ceroni, P.; Paolucci, F.; Cozzi, P. G.; Scattolin, T.; Demitri, N.; Negri, F.; Gualandi, A.; Aliprandi, A.; Valenti, G. Tunable Electrochemiluminescence of TADF Luminophores: Manipulating Efficiency and Unveiling Water-Soluble Emitters. *Chem. Sci.* **2024**, *15*, 17892–17899.
